# The Epidemiology and Economic Burden of Obesity and Related Cardiometabolic Disorders in the United Arab Emirates: A Systematic Review and Qualitative Synthesis

**DOI:** 10.1155/2018/2185942

**Published:** 2018-12-03

**Authors:** Hadia Radwan, Rami A. Ballout, Hayder Hasan, Nader Lessan, Mirey Karavetian, Rana Rizk

**Affiliations:** ^1^Department of Clinical Nutrition and Dietetics, College of Health Sciences, Research Institute of Medical & Health Sciences (RIMHS), University of Sharjah, P.O. Box 27272, Sharjah, UAE; ^2^Faculty of Medicine, American University of Beirut, Beirut, Lebanon; ^3^Imperial College London Diabetes Centre, P.O. Box 48338, Abu Dhabi, UAE; ^4^Department of Health Sciences, College of Natural and Health Sciences, Zayed University, P.O. Box 144534, Dubai, UAE; ^5^INSPECT-LB, Institut National de Santé Publique, d'Épidémiologie Clinique et de Toxicologie, Faculty of Public Health, The Lebanese University, Beirut, Lebanon; ^6^Department of Health Services Research, Maastricht University, 6200 MD Maastricht, Netherlands

## Abstract

**Background:**

Noncommunicable diseases (NCDs) are considered as a global health problem and considered as a public health priority with the more considerable increasing trend of obesity and cardiometabolic disorders rates in the Middle Eastern countries. This systematic review aims at assessing the prevalence, incidence rates, and trends, as well as the cost of obesity and related cardiometabolic disorders in the United Arab Emirates (UAE).

**Methods:**

A highly sensitive strategy was used to retrieve original observational studies, addressing the epidemiology and cost of obesity and related cardiometabolic disorders in the UAE, irrespective of nationality (nationals and expatriates). The search was conducted on April 4, 2017, within numerous electronic databases and the grey literature. Standardized and validated methods were used for data extraction and analysis as well as quality assessment.

**Results:**

6789 records were retrieved, of which 36 were deemed eligible. High prevalence rates were reported for obesity, diabetes, hypertension, and metabolic syndrome in all studies. However, the definitions and methods employed by the studies were highly variable. The risk of bias in the epidemiological studies ranged between low and medium. Only one study reported the cost of illness for diabetes. In this study, the estimated cost per patient was $2,015 (adjusted to the year 2015), and it became twofold and sixfold higher in patients with microvascular and macrovascular complications, respectively.

**Conclusions:**

Obesity and related cardiometabolic disorders are highly prevalent in the UAE, but quoting a precise prevalence for them is difficult given the methodological heterogeneity of the epidemiological studies addressing them. Nonetheless, we detected a 2-3-fold increase in the prevalence of overweight and obesity in the UAE between 1989 and 2017. It is hopeful that this systematic review will provide an insight into direct future studies, especially longitudinal studies exploring obesity and cardiometabolic risks and their costs.

## 1. Introduction

The global rise in the prevalence rates of obesity, metabolic syndrome, and diabetes has been linked to recent lifestyle changes occurring during the past few decades, with the Middle East in general and the United Arab Emirates (UAE) in particular demonstrating dramatic increases in those rates [1]. The UAE has witnessed an economic boom that was accompanied by rapid urbanization and an influx of expatriate workforce [1]. The combination of these factors meant a modern, fast-paced, and technology-driven lifestyle which, in turn, resulted in a reduction in occupational, domestic, and leisure-time physical activity [2, 3], as well as an excessive consumption of calorie-dense, processed, and prepackaged meals of poor nutritional value [3].

The abovementioned risk factors, in addition to the high incidence of tobacco use in the UAE, possibly explain the witnessed increase in obesity and cardiometabolic disorder rates in the country [4, 5], resulting in increased demand for disease-specific health services. A recent systematic review on the UAE's health status ranks cardiovascular diseases as a top public health priority for the country, attributing to it the majority of noncommunicable disease- (NCD-) related mortalities [6]. This overlaps with the global epidemiology of NCDs, among which cardiovascular diseases also rank first in terms of incidence and mortality [7]. In fact, the UAE's 2021 vision is to adopt the number of deaths due to cardiovascular diseases per 100,000 population, the prevalence of diabetes, and the prevalence of obesity amongst children as its primary national performance indicators for a desired world-class health care, in order to guide targeted interventions and public health efforts [8].

Therefore, the following systematic review provides a current evidence-based assessment of the epidemiology and economic burden of obesity and cardiometabolic disorders in the UAE, given the lack of such an assessment. We hope that our reported data provide an up-to-date epidemiological profile (prevalence, incidence, and trends) for the country with regard to cardiometabolic disorders and their associated costs and that this would eventually guide public health policy-makers in prioritizing and allocating resources properly for managing and preventing those diseases [9]. We also hope that our review highlights the current gaps in relevant research in preparation for subsequent research efforts.

## 2. Methods

We followed the standardized methods outlined by Moher et al. in the PRISMA 2010 group in conducting and reporting our systematic review [10]. However, we drafted a priori a protocol for the review and published it in the International Prospective Register of Systematic Reviews (PROSPERO) (CRD42016035747).

### 2.1. Database Search

We searched MEDLINE, PubMed, Embase, Cumulative Index to Nursing and Allied Health Literature (CINAHL), Index Medicus for the Eastern Mediterranean Region (IMEMR), ProQuest Dissertations & Theses Database (PQDT), Open Access Theses and Dissertations (OATD), and Web of Science for studies addressing the epidemiology and costs of cardiometabolic disorders in the UAE, using an extensive and highly-sensitive search strategy (Appendix A-a in Supplementary Materials). We replicated the search employing appropriate and specifically adapted vocabulary for each of the databases searched in order to retrieve all potentially eligible references. The initial search was conducted on June 19, 2015, and updated on April 4, 2017, to ensure that our review is current.

### 2.2. Searching Other Sources

In addition to the above databases, we searched the grey literature, the International Diabetes Federation (IDF), and primarily the World Bank websites for additional eligible studies. We also contacted prominent scholars and experts in the field from the UAE to inquire about any ongoing relevant studies not published yet.

It is worth mentioning that, in our original search, we aimed at finding studies that address the epidemiology and costs of either cancer and/or cardiometabolic disorders in the UAE, which are the top two incident NCDs within the UAE and thus the lead priorities on its public health agenda [6]. However, given the difference between the two diseases with regard to their underlying determinants (i.e., risk factors) and patient profiles and in order to generate disease-specific epidemiology and/or cost data for specialized policy-makers and researchers in each field, we opted to report the data for each of them in a separate systematic review. Nonetheless, both reviews were registered with the same protocol (CRD42016035747).

### 2.3. Inclusion and Exclusion Criteria

As outlined in our protocol, studies eligible for inclusion had to meet the following criteria:

- Be original studies (e.g., editorials, case reports, case series, and reviews were excluded)
- Have an observational design: cohort or cross-sectional studies for epidemiological outcomes and economic models and cross-sectional or longitudinal studies for cost analyses
- Address cardiometabolic disorders defined as cardiovascular diseases of all types, type 2 diabetes, and/or metabolic syndrome including its primary components of obesity, insulin resistance, dyslipidemia, and/or hypertension
- Report data specific for the UAE's citizens irrespective of their nationality (nationals and/or expatriates), sex (women and/or men), or age (children and/or adults)
- Written in English, Arabic, or French, irrespective of publication status (published versus unpublished) or date (i.e., no time limit)

### 2.4. Selection of Studies

Three reviewers (RB, HR, and RR), assisted by a reference manager (EndNote©), looked for potentially eligible studies by screening the titles and abstracts of the records retrieved by the search. After conducting a prior calibration exercise to ensure inter-reviewer screening consistency, two pairs of authors (RB/RR and HR/HH) individually and in pairs retrieved and evaluated the full texts of one-half of the references deemed eligible before (i.e., during the title and abstract screening phase) for inclusion in data extraction. A screening tool was developed by the three reviewers (RB, HR, and RR) and pretested through a calibration exercise prior to the actual full-text screening. Disagreements were resolved through discussion with a third reviewer.

### 2.5. Data Extraction

The two reviewers in each pair (RB/RR and HR/HH) individually and in pairs extracted relevant data from the included studies, consulting a third reviewer whenever they disagreed. As set in our protocol, the reviewers performed a qualitative (i.e., narrative) synthesis of the data extracted from the included studies, given that quantitative synthesis (i.e., meta-analysis) is not possible due to the epidemiological nature of the data extracted.

### 2.6. Risk of Bias Assessment

The reviewers used the tool developed and validated by Hoy et al. [11] to assess the risk of bias in the included studies. The tool comprises 10 items that address both the external and internal validity of each study, with an additional item that provides an overall summary of the risk of bias in the study. Each item is categorized as having “high risk,” “intermediate risk,” or “low risk” of bias, with the overall risk of bias being lower when more criteria (i.e., items) are adequately met. A high risk of bias was assigned to studies with unclear or poor reporting of a particular item. Finally, a study was considered to have an overall high risk of bias when it met less than 5 criteria, moderate risk of bias when it met 5 to 7 criteria, and low risk of bias when it met 8 or more criteria.

## 3. Results

Our initial search retrieved 6789 records, of which only 30 were deemed eligible. The updated rerun of the search retrieved 6 additional eligible studies, yielding a total of 36 studies that were eligible and included in data extraction and synthesis (Appendix A-b in Supplementary Materials).

### 3.1. Characteristics of Included Studies

Tables 1 [Table tab2]–3 summarize the characteristics, results, and overall risk of bias in the included epidemiological studies (*n*=35).  Table 4 summarizes the characteristics and results of the single study identified that addresses the cost of illness (COI) for diabetes [12]. Included studies addressed various cardiometabolic disorders, including overweight/obesity (*n*=19), metabolic syndrome (*n*=3), dyslipidemia (*n*=1), hypertension (*n*=2), diabetes (*n*=4; 2 prevalence, 1 incidence, and 1 cost), and multiple cardiometabolic disorders at once (*n*=7). Only one study was a retrospective cohort [13], with the rest being cross-sectional studies (*n*=35). All included studies were published between 1995 and 2016 (1990–1999: *n*=6; 2000–2009: *n*=15; and after 2009: *n*=15), reporting data collected between 1989 and 2015. Half of the included studies reported data specific to Emiratis (*n*=18), and two-thirds (*n*=24) reported data for both sexes. Only six studies were conducted at the national level [3, 14–18], with the rest being emirate-specific. Finally, more than half of the included studies used a random sample (*n*=20), seven recruited a convenient sample, four adopted exhaustive sampling (census), and one included a purposive sample. Four studies did not report their sampling method. It is worth noting that the included studies remarkably varied in their recruited sample size, ranging from 98 [19] up to 173,501 [20] participants.

## 4. The Epidemiology of Cardiometabolic Disorders in the UAE

### 4.1. Overweight and Obesity

#### 4.1.1. Paediatric Population

Abdulrazzaq et al. (1991-1992) provided the earliest and most exhaustive national-level estimates of the prevalence of overweight and obesity in the Emirati paediatric population, reporting rates of 13% for boys below the age of 5 and 6% for their female counterparts [16]. The study reports that these figures gradually increase with age, peaking at 18 years. Three other studies address more recent national estimates for overweight and obesity in Emirati schoolchildren and adolescents, with a 10-year interval difference between the two older studies (1998-1999) [22] and the most recent one (2009-2010) [3]. Despite reporting similar overweight estimates of about 19% for each of the boys and the girls and no major differences in sex-specific rates, the two older studies [15, 22] remarkably differed in their reported overall obesity rates (7% [22] versus 13% [15]). In contrast, the more recent study highlights the emergence of sex-specific differences, particularly in overweight rates in children aged 6 to 10 (24% for girls versus 9% for boys) and obesity rates in adolescents aged 11 to 18 (20% for girls versus 12% for boys) [3]. The study reports an overall prevalence of 40% of overweight and obesity for Emirati female children compared to 25% only for their male counterparts [3]. The same was reported in adolescents. On the contrary, at the emirate-specific level, Fujairah seems to harbour the highest rates of overweight and obesity, reporting a striking 30% prevalence among its Emirati schoolgirls [21]. Two other studies assessed the prevalence of overweight and obesity in Emirati schoolchildren in Ras Al Khaimah, another emirate, with more than a decade as the time difference between the older study [14] and the recent one [23]. Direct comparison of the results of these two studies [14, 26] is challenging given their use of different definitions for overweight and obesity and the inclusion of children of different age groups. However, despite those differences, the rates of overweight and obesity reported by the older study compared to the recent one show a tremendous increase over time, almost doubling for overweight (9% versus 17%, respectively) and more than doubling for obesity (8% versus 20%, respectively). As for Abu Dhabi, two recent studies report similar rates of approximately 34% of overweight and obesity prevalence among both Emirati and non-Emirati schoolchildren [25, 35]. These studies have also employed different disease definitions and included children of different age groups, which prevented us from providing conclusive evidence through directly comparing their findings. However, the two studies seem to be in agreement with regard to their reported sex-specific rates, highlighting a greater proportion of overweight and obesity in boys compared to girls in Abu Dhabi. In contrast, Dubai shows almost equal rates of obesity in the two sexes (girls 21% and boys 22%), yet higher rates of overweight in boys (19%) compared to girls (13%) [24]. Conversely, Sharjah shows identical overweight rates among its boys and girls (11%), with more than triple the rate of obesity in its boys (23%) compared to girls (7%) [26].

#### 4.1.2. University Students

All studies addressing the rates of cardiometabolic disorders among university students were conducted at the UAE University in Al Ain, Abu Dhabi. They report varying rates of overweight and obesity across female university students, ranging between 29% [32] and 46% [30]. In contrast, only one study tackled male university students and reported an obesity rate of 35% among them [29] without reporting their overweight rates.

#### 4.1.3. Adults

A study conducted at the national level between 1999 and 2000 reported prevalence rates of 40% and 30% for overweight and obesity, respectively, in Emirati and non-Emirati adults combined [14]. In contrast, a more recent study reports a prevalence of 42% for overweight and 20% for obesity in 2012 among the same abovementioned population [42]. However, the latter study recruited a convenient sample of Emirati and non-Emirati adults in 5 of the UAE's 7 emirates, making the deduction of temporal trends somewhat unreliable. Regarding sex-specific rates in the adult population, a recent national study shows that nearly two-thirds of adult Emirati females carry excess body weight (31% overweight and 34% obese) [3], yet it does not report any estimates for males. However, an earlier study found remarkable differences in sex-specific rates of obesity, reporting a prevalence of 47% in women compared to 28% in men [43]. The same study reported an overall estimate of 37% for overweight and obesity in Emirati adults residing in Abu Dhabi [43]. Likewise, in a study reporting emirate-specific rates, it was estimated that around three-quarters of the Emirati adults residing in Abu Dhabi carry excess body weight, with 34% and 41% of them being overweight and obese, respectively [20].

### 4.2. Metabolic Syndrome

The national estimates of metabolic syndrome among Emirati and non-Emirati adults were 38% and 41%, respectively, in 1999-2000, using the National Cholesterol Education Adult Panel III (NCEP) and IDF definitions [17]. Interestingly, regardless of the definition used, the rate of metabolic syndrome appears to be higher in females compared to males (47% and 46% versus 32% and 33%, according to the NCEP and IDF definitions, respectively). Particularly, the two components that were more accentuated in females compared to males in the NCEP definition were a low level of high-density lipoprotein cholesterol (HDL-C) (54% of cases) and a high fasting plasma glucose (51% of cases) [17]. As for schoolchildren in Abu Dhabi, around 13% of those aged 12 to 18 years suffer from metabolic syndrome as defined by the IDF, although with a greater prevalence in boys (22%) than girls (4%) [35]. According to that study, the most common components of the IDF definition that remarkably differed between the two sexes were a low HDL-C and an elevated waist circumference. Finally, among university students, only 7% of the female Emirati students attending the UAE University were found to have metabolic syndrome [36], with 38% of the study's participants satisfying at least one component of the IDF definition of metabolic syndrome [36].

### 4.3. Dyslipidemia

One study particularly addressing dyslipidemia, defined as a total cholesterol >240 mg/dL, reports prevalence estimates of 19% in Emirati and non-Emirati Arabs compared to 11% in non-Arabs [29]. The study also shows increasing rates with advancing age (23% in those > 51 years old versus 17% in younger participants). A more recent study addressing dyslipidemia estimates in a convenient sample of adults from 5 emirates (Dubai, Abu Dhabi, Sharjah, Fujairah, and Ras Al Khaimah) reports a 69% overall rate of elevated total cholesterol (≥200 mg/dl) or reduced HDL-C level (<40 mg/dl) [42]. The study was conducted as part of assessing the cardiometabolic status of those emirates [42]. Two other studies conducted 5 years apart (2004-2005 [43] and 2009-2010 [20]) report comparable rates of dyslipidemia among the Emirati citizens of Abu Dhabi (59% and 51%, respectively), despite using different disease definitions. Interestingly, Hajat et al. highlight sex-based differences in the rates of dyslipidemia (defined as LDL-C ≥ 4.1 mmol/L or HDL-C ≤ 1.0 mmol/L), reporting a prevalence of 57.7% in men compared to 33.9% in women [20].

### 4.4. Impaired Fasting Glucose, Prediabetes, and Diabetes

The sole study conducted at the national level in the UAE was between 1999 and 2000, reporting a prevalence of 21% for diabetes and 7% for impaired fasting glucose (IFG) [18]. A decade later, another study addressing the prevalence of diabetes in a convenient sample from five emirates reported an increase in prevalence to 32% [42]. However, Abu Dhabi remains the only emirate in which diabetes prevalence can be trended, given the multiple studies on the topic since 1989 [20, 40, 41, 43]. Of these studies, the oldest reports an age-standardized rate (ASR) of 6% for diabetes within a purposive sample of Bedouin Emirati adults between 1989 and 1990 [40]. The subsequent study, conducted more than a decade later, reports an increase in the ASR of diabetes to 17%, with a concomitantly high ASR for prediabetes (20%) [41]. Another study conducted afterward reports an even higher estimate for diabetes (23%) [43]. Finally, the most recent of those studies states that more than half of the Emirati adults of Abu Dhabi suffer from dysglycemia, reporting increased estimates of both diabetes (24.6%) and prediabetes (29.5%), with no significant differences across the corresponding sex-specific rates [20]. Thus, by comparing the diabetes estimates reported in those studies in their respective chronological order, we clearly recognize an uptrend in the prevalence of diabetes in Abu Dhabi throughout the years. On the contrary, only one study addresses the incidence of diabetes in Ajman, reporting an overall rate of 4.8 per 1,000 person-years (PY), with its highest rates being seen in those aged 55 to 59 years (23.4 and 32.4 per 1,000 PY for men and women, respectively) [13]. That study also shows that the incidence rates of diabetes among women remarkably increase after the age of 40, becoming almost double the corresponding rates for men (6.3 versus 3.3 per 1,000 PY, respectively).

### 4.5. Prehypertension and Hypertension

One study addressed the prevalence of hypertension in 5 emirates and reported an overall estimate of 31% for hypertension prevalence in adults [42]. Hypertension was defined as having a history of known and treated hypertension, having a systolic blood pressure equal to or above 140 mm·Hg, or having diastolic blood pressure equal to or above 90 mm·Hg [42]. At an emirate-specific level, only one study addressed the prevalence of hypertension in the Emirati adults of Sharjah, reporting an ASR of 37% [39], which is much higher than rates reported by the two studies addressing hypertension prevalence in Abu Dhabi's Emirati adults (21% in the older study [40] and 29% in the more recent one [25]). However, despite the increase in the overall estimates of hypertension in Abu Dhabi reported by the two latter studies (from 21% to 29%), it is difficult to accurately state whether this increase truly reflects an uptrend in hypertension prevalence, given the differences in the methodologies adopted by the two studies and the definitions used for elevated blood pressure. Interestingly, however, a higher prevalence of hypertension is noted in males compared to females in all three aforementioned studies [20, 39, 43]. As for the paediatric population, only one study addressed the prevalence of hypertension and reported that more than one-quarter of the Emirati schoolchildren residing in Abu Dhabi have elevated blood pressure (11% prehypertensive and 17% hypertensive, with a predominance of systolic hypertension in the hypertensive children) [42].

### 4.6. Cardiometabolic Disorders in the Working Class

Only two of the included studies addressed the prevalence of cardiometabolic disorders in adult employees in particular [45, 46]. The older study reported a prevalence of 74% for dyslipidemia and 68% for obesity in both national and expatriate male workers [45]. In contrast, the more recent study compared the rates of cardiometabolic disorders in oil and gas company male workers of Abu Dhabi prior to employment to rates seen at postemployment periodic health evaluations [46]. It demonstrated an increase in hypertension and diabetes rates by about 20% and 9%, respectively, with a decline in obesity rates by almost one-half (from 16.6% to 8.6% from preemployment to postemployment), all occurring within 3 years of employment [46].

Assessment of the included studies revealed that one-third of them had a medium overall risk of bias (11/35), and the remaining ones had a low risk of bias (24/35). The most commonly encountered defects in the included studies were failing to recruit truly representative samples of the target population (*n*=21), employing a nonrepresentative sampling frame (*n*=11), and recruiting nonrandom (i.e., convenient) samples (*n*=9).  Table 3 summarizes the risk of bias assessment for the included studies.

### 4.7. Cost of Illness (COI) for Diabetes

Only one of the included studies addressed the COI for diabetes, one of the cardiometabolic disorders of interest to us [12]. This cross-sectional study, which was conducted in 2004, adopted a health care-payer perspective that accounted for all the direct medical costs of diabetes for Emirati and non-Emirati patients attending the outpatient clinics at the two major referral hospitals (i.e., Al Ain and Tawam) regardless of age or sex.  Table 4 summarizes the characteristics and results of the study. In short, using a macro-cost approach, the study estimates the annual cost of diabetes to be around US$1,605 for patients with complication-free cases ($2,015 adjusted for the year 2015). In contrast, this value almost doubles for patients with microvascular complications, increases by more than sixfold for those with macrovascular complications, and increases by more than ninefold for those with concomitant micro- and macrovascular complications [12]. However, the study's limited reporting of cost estimates, its failure to identify major uncertainties or perform any sensitivity analyses, and its insufficient documentation and justification for its reported estimates render its overall methodological quality of the suboptimal level (Appendix B in Supplementary Materials).

## 5. Discussion

This systematic review provides an overview of the prevalence of major cardiometabolic disorders in the UAE, namely, overweight and obesity, metabolic syndrome, dyslipidemia, diabetes, and hypertension.

The last study conducted to date at the national level in the UAE addressing overweight and obesity prevalence in Emirati and non-Emirati adults reported that the overall prevalence rates for overweight and obesity are 40% and 33%, respectively, showing that almost three-quarters of the UAE's adults (73%) have a body mass index (BMI) ≥25 kg/m^2^ [47]. This somewhat mirrors the combined overall rates for overweight and obesity seen in the United States during the same period (64.5%) [42]. However, a more recent study by Yusufali et al. estimates that 62% of the UAE's adults have a BMI ≥25 kg/m^2^, based on data collected from five emirates in 2012, suggesting a slight drop in obesity and overweight rates [42]. In fact, the overall estimates of overweight and obesity reported by the study (42% and 20%, respectively) [42] are clearly lower than those reported for earlier years [18]. However, it remains rather imprecise to deduce any trends in overweight or obesity rates by simply comparing the two studies due to their major differences with regard to design, sample recruitment, and specimen analysis. Nonetheless, the UAE still appears to be doing better than several of its neighbouring countries with regard to its overweight and obesity rates, such as Oman or Saudi Arabia (ASR of 64.7% and 63.6%, respectively) [48, 49].

The World Health Organization's (WHO) more recent report on NCD prevalence demonstrates high rates of overweight and obesity in the UAE's adults, reporting similar estimates for the two sexes (75.8% in female and 73.1% in male) [48]. Such national-level estimates in the UAE mirror the combined rates reported by one of our included studies for overweight and obesity in each sex among the Emirati adults residing in Abu Dhabi (overweight ASR: 34.4% in men and 33.6% in women, added to obesity ASR: 41.5% in men and 40.7% in women) [20]. This is possibly due to Abu Dhabi's (UAE capital) dense population and prominent role in the country's economic and political status, somewhat rendering it a miniature representative version of the entire country. The aforementioned study highlights that while the two sexes in the UAE have almost equal rates of overweight and obesity, their counterparts in the United States (US) have an almost 10% difference in these rates (75% in men versus 66.5% in women) [50]. More extremely, Oman shows an almost 10-fold higher ASR for obesity in its women compared to men (44.3% versus 4.7%, respectively) [51]. This is likely because the rates of overweight and obesity across the two sexes remarkably differ by region, depending primarily on differences in social and cultural values, as highlighted in the 2011 global survey [52]. As for trending the rates of overweight and obesity in the UAE, only one study included in our review reports an observed uptrend of about 35% in the Emirati women residing in the Al Ain city of Abu Dhabi between 2000 and 2004, without reporting any trends for rates in men [34]. In fact, none of the included studies addresses the trends in overweight and obesity rates in men, another issue highlighted by the global survey as well [52].

As for the metabolic syndrome, only one included study addressed the national prevalence of metabolic syndrome among the UAE's adults between 1999 and 2000, reporting overall rates of 38% (as per NCEP definition) and 41% (IDF definition). The study also highlights remarkable differences in the sex-specific estimates of metabolic syndrome, reporting higher rates for women (47% and 46% as defined by the NCEP and IDF, respectively) compared to men (32% and 33% as defined by the NCEP and IDF, respectively) [17]. In contrast, the overall prevalence of metabolic syndrome in Oman as defined by the IDF was only 19.8% in 2006, almost half of that in the UAE [53]. Interestingly, however, the Omani study highlights even more pronounced differences in the sex-specific ASRs for metabolic syndrome (18.4% in men versus 40% in women) [53] compared to those in the UAE. This clearly demonstrates much lower rates of metabolic syndrome in Omani males compared to their UAE counterparts (18.4% versus 33%), as opposed to the almost equal rates seen across Omani and UAE females (40% and 46%, respectively) [17]. In contrast, Saudi Arabia's overall ASR for metabolic syndrome was reported to be 39.3% between 1995 and 2000, as defined by NCEP [54], which is almost similar to the UAE's 1999-2000 estimate (41%). Likewise, the sex-specific ASRs for metabolic syndrome reported for the two sexes in Saudi Arabia also mirror those of men and women in the UAE using the NCEP definition (37.2% and 42% in Saudi Arabia's men and women, respectively, versus 32% and 47% in the UAE's men and women, respectively) [54].

Regarding dyslipidemia, there is an uptrend in the overall dyslipidemia rates in the UAE in recent years. A 2012 study included in our review addressed Emirati adults residing in Abu Dhabi and reported an ASR of 50.7% for dyslipidemia [20]. Another study conducted 3 years later reported a higher overall estimate of 68.5% for dyslipidemia prevalence among the adults in five of the UAE's emirates [42]. However, while the former study highlights remarkable differences in the sex-specific rates of dyslipidemia, reporting crude rates of 57.7% in men versus 33.9% in women [20], the more recent study does not break its overall estimate into sex-specific rates, making us unable to trend the sex-specific rates of dyslipidemia in recent years. In contrast, despite its higher rates of overweight and obesity, Saudi Arabia paradoxically has a lower overall rate of dyslipidemia (44%) compared to the UAE (68.5%), as reported by a national Saudi study published in the same year [55]. As for the sex-specific rates of the individual components of dyslipidemia in Saudi Arabia, the latter study reported similar rates of hypercholesterolemia and elevated low-density lipoprotein cholesterol (LDL-C) levels in both sexes (18.7% and 30.7% in men and 19.9% and 29.8% in women, respectively), with significantly higher rates of low HDL-C levels in men compared to women (33.7% versus 17.7%, respectively; *p* value = 0.001) [55]. A national study conducted in Kuwait, another neighbouring country to the UAE, addressed the temporal trends in hypercholesterolemia rates defined according to the NCEP criteria from 1998 through 2009 [49]. The study clearly reported a progressive increase in the sex-specific rates of hypercholesterolemia, reaching 56% and 53.6% in men and women, respectively, in 2007, followed by a significant drop thereafter to 33.7% and 30.6%, respectively, in 2009 [49]. However, given that the prevalence rates of hypercholesterolemia, high LDL-C levels, or low HDL-C levels were collectively reported as “dyslipidemia” in the studies conducted in the UAE, we were unable to compare the specific rates of each of those lipid entities in the UAE to their corresponding estimates in nearby countries.

Only one included study addressed the prevalence of diabetes at the national level in the adult population of the UAE, reporting an overall ASR of 21% for diabetes and 7% for impaired fasting glucose between 1999 and 2000 [18]. These values are clearly higher than those reported in the US during the same time interval (1999-2000; 8.6% and 6.2%, respectively) [56], highlighting remarkably higher rates of diabetes in the UAE compared to the US (21% versus 8.6%, respectively), despite similar rates of impaired fasting glucose (7% versus 6.2%, respectively). However, both studies report similar ASR of diabetes in men and women, although the overall rates reported in the UAE (20.4% and 22.3%, respectively) are higher than those seen in the US (9.3% and 8.1%, respectively) [56]. In contrast, impaired fasting glucose rates are remarkably higher in women compared to men in the UAE (7.2% versus 4.5%, respectively) [18], which is the complete opposite of what is seen in the US where the ASR for impaired fasting glucose in men is almost double that in women (8.3% versus 4.5%, respectively) [56].

Furthermore, a more recent study included in our review reports ASR of 29.5% and 24.6% for prediabetes and diabetes, respectively. Taken together, these rates suggest that more than half of the Emirati adults of Abu Dhabi suffer from impaired glycemic control [20]. However, no differences were noted across the sex-specific rates reported by that study. These findings should alarm public health policy-makers in the UAE and should highlight the need for prompt intervention to curb the high rates of impaired glycemic control in the UAE in order to prevent further progression into diabetes [57]. Additionally, it is important to draw attention to the 2011 IDF statistics which rank Kuwait first in the world with regard to its high national prevalence of type 2 diabetes (21.2%), followed by Qatar (20.1%), Saudi Arabia (20.0%), and Bahrain (19.8%), all of which happen to fall in the immediate vicinity of the UAE [58]. This clearly shows a regional pooling of diabetes along the western banks of the Arabian Gulf, possibly due to underlying common genetic and/or ethnic backgrounds of the citizens in those countries, added to the possible roles of their similar environmental, social, and dietary factors as well.

Hypertension also falls among the UAE's predominant cardiometabolic disorders, with all relevant studies conducted to date reporting emirate-specific estimates rather than national ones [20, 39, 42]. Thus, we refer to the WHO 2014 global status report on NCDs that found an overall ASR of 26.3% for elevated blood pressure in the UAE and almost similar rates across the two sexes (27.5% in men and 23.3% in women) [48]. In contrast, Dubai Health Authority (DHA) and Dubai Statistics Center (DSC) collaboratively gathered data on the prevalence of various cardiovascular risk factors including hypertension between 2014 and 2016 and reported an overall estimate of 18.9% for hypertension prevalence among the Emirati citizens of Dubai [59]. The study also reports similar rates across the two sexes (20% in men and 18% in women). Another emirate-specific study run by the Health Authority of Abu Dhabi (HAAD) 3 years earlier than the latter study reports an overall prevalence of 17% for hypertension among Abu Dhabi's Emirati citizens compared to double that prevalence (35%) in its non-Emirati citizens [60]. However, contrary to the similar sex-specific rates of hypertension reported in the Dubai study [59], HAAD highlighted a significant difference in the rates of hypertension across the two sexes in Abu Dhabi, reporting a twofold higher rate in Emirati men (24%) compared to women (12%) [60]. Saudi Arabia shares similar estimates with its UAE neighbour, reporting an almost identical overall prevalence rate of hypertension among its adults (25.5%) in 2011 [61]. In contrast, Oman, another country bordering the UAE, reports almost double that rate for adult hypertension, giving a strikingly high estimate of 41.5% in 2015 [62]. However, Yemen, a country bordering each of Saudi Arabia, Oman, and the UAE, reported a remarkably much lower overall ASR of hypertension than any of its neighbours in 2013 (7.7%) [63]. Such findings pose a question on the factors that play a major role in the development of hypertension, given that its rates in geographically proximal, culturally similar, and ethnically related countries are remarkably different as shown previously. This also contradicts with the apparent pooling of diabetes in that same geographical area, as highlighted earlier.

Our review had some limitations worthy of being addressed. For instance, costs associated with cardiometabolic disorders in the UAE were not properly reported due to the lack of relevant studies. Moreover, the overall quality of some national studies limited our ability to provide conclusive evidence about the trend of cardiometabolic disorders.

## 6. Conclusions

However, our systematic review's major strength is its highly sensitive search strategy that possibly covered all relevant and intended literatures adequately. Moreover, our adherence to standardized and validated methods in conducting the review [10], our transparency in disseminating our search strategy (Appendix A in Supplementary Materials), and our use of standardized and previously tested data extraction and risk of bias assessment tools further support the review, increasing confidence in its reported findings. We are also the first team of researchers to conduct a systematic review of the epidemiology of obesity and cardiometabolic disorders in the UAE, setting grounds for subsequent researchers to build on. Thus, the ultimate aim of this review was to simply provide insights into the current prevalence rates and associated costs of cardiometabolic disorders in the UAE, making local decision-makers better informed and therefore capable of altering and tailoring future health policies accordingly.

## Figures and Tables

**Table 1:** Characteristics of included epidemiological studies

	Author and year	Studied disease (criteria)	Study type	Participant characteristics	Sampling
Obesity in children	Al Hourani et al.. (2003) [21]	At risk for overweight: BMI ≥ 85–95th percentiles for age and sex Overweight: ≥95th percentile for age and sex (NHANES reference data)	Cross-sectional Period: October 1998–April 1999	Emirate: Abu Dhabi (43.2%), Sharjah (19.2%), Dubai (16.3%), Ras Al Khaimah (16.1%), and Fujairah (5.2%) Nationality: Emirati Gender: female schoolchildren (public) aged 11–18 years	Unclear Sample #: 898
	Al Haddad et al. (2005) [22]	Overweight: BMI ≥ 25 kg/m^2^ and <30 kg/m^2^ Obesity: BMI ≥30 kg/m^2^ (Cole et al. International Standards for overweight and obesity)	Cross-sectional Period: October 1998–April 1999	Emirate: All (national) Nationality: Emirati Schoolchildren aged 4–18 years	Multistage stratified cluster sampling; stage 1: educational districts; stage 2: schools by PPS; stage 3: all Emirati students Sample #: 15,989
	Malik and Bakir (2007) [15]	Overweight: BMI >25 kg/m^2^ Obesity: BMI >30 kg/m^2^ (IOTF classification)	Cross-sectional Period: October 1998–April 1999	Emirate: All (national: Abu Dhabi: 47.2%; Abu Dhabi (Al Ain): 34.5%; others: 18.3%) Nationality: Emirati: 48%; others: 52% Gender: boys: 49.6%; girls: 50.4% Schoolchildren (public and private) aged 5–17 years	Two-stage PPS cluster random sampling: stage 1: schools (categorized according to size, gender, ethnic mix, type, and area of residence; randomization method not detailed); stage 2: one or more whole class per each school grade (25 children) Sample #: 4,381
	Abdulrazzaq et al. (2011) [16]	Overweight: (1) Under 5 years: ≥1 SD (equivalent to BMI 25 kg/m^2^ at 19 years) (WHO criteria) (2) 18 years old: IOTF guidelines (3) Other age groups (NR) Obesity: (1) Under 5 years: ≥2 SD (equivalent to BMI 30 kg/m^2^ at 19 years) (WHO criteria) (2) 18 years old: IOTF guidelines (3) Other age groups (NR)	Cross-sectional Period: 1991-1992	Emirate: All (national) Nationality: Emirati Age: 0–18 years	Multistage stratified random sampling (not detailed) Sample #: 20,494
	Al Haddad et al. (2000) [14]	Overweight: BMI ≥85th and <95th percentiles for age and sex Obesity: BMI ≥95th percentile for age and sex or BMI ≥30 kg/m^2^, whichever is smaller (NHANES reference data)	Cross-sectional Period: NR	Emirate: Ras Al Khaimah Nationality: Emirati Gender: girls: 56.1%; boys: 43.9% Schoolchildren aged 6–16 years	Unclear Sample #: 4,075
	Al Blooshi et al. (2016) [23]	Overweight, obesity, and extreme obesity: IOTF (1) Overweight: BMI ≥25 kg/m^2^ equivalent and <30 kg/m^2^ equivalent (2) Obesity: BMI ≥30 kg/m^2^ equivalent WHO (1) Overweight: BMI for age ≥85th percentile and <95th percentile (2) Obesity: BMI for age ≥95th percentile CDC (1) Overweight: BMI for age ≥85th percentile and <95th percentile (2) Obesity: BMI for age ≥95th percentile	Cross-sectional Period: 2014-2015	Emirate: Ras Al Khaimah Nationality: Emirati: 92%; others: 8% Gender: girls: 51%; boys: 49% Mean age: 10.4 (3.9) (range: 3–18 years) Schoolchildren (public)	Exhaustive (all governmental schools in Ras Al Khaimah, with assigned nurses or where height/weight measurements are directly supervised) Sample #: 29,410
	Bin Zaal et al. (2009) [24]	Overweight: 85th to <95th BMI percentiles Obesity: ≥95th BMI percentile (WHO, 1995)	Cross-sectional Period: NR	Emirate: Dubai Nationality: Emirati Gender: girls: 51%; boys: 49% Age range: 12–17 years Preparatory and secondary school students	Multistage stratified random sampling (stratified by sex and school type: preparatory and secondary; randomization method not detailed) Sample #: 661
	Al Junaibi et al. (2013) [25]	Overweight: 85th < BMI <95th CDC percentile for age and sex Obesity: BMI ≥95th CDC percentile for age and sex	Cross-sectional Period: January–December 2011	Emirate: Abu Dhabi Nationality: Emirati: 71.9%; others: 28.1% Gender: girls: 48.9%; boys: 51.1% Schoolchildren (public) aged 6–19 years	Two-stage stratified sampling by gender: stage 1: schools; stage 2: students by PPS Sample #: 1,440
	Musaiger et al. (2012) [26]	Overweight: IOTF reference standard Obesity: IOTF reference standard	Cross-sectional Period: March 2010–January 2011	Emirate: Sharjah Nationality: NR Gender: boys: 51.9%; girls: 48.1% Mean age: boys: 16.41 (0.93); girls: 16.54 (0.99) (range: 15–18 years) Students in secondary schools (public)	Multistage stratified random sampling: stage 1: administrative regions, stage 2: schools by PPS to administrative regions, and stage 3: classes (simple random method) Sample #: 505

Obesity in university students	Amine and Samy (1996) [27]	Overweight: 110–120% of the reference value for standard weight for height tables issued by the Nutrition Institute in Cairo, Egypt Obesity: >120% of the reference value for standard weight for height tables issued by the Nutrition Institute in Cairo, Egypt	Cross-sectional Period: NR	Emirate: Abu Dhabi (Al Ain) Nationality: Emirati (Abu Dhabi: 40.3%, Dubai: 17.5%, Sharjah: 19.3%, Ras Al Khaimah: 14%, and others: 9%) Gender: female Students in UAE University	Stratified (according to the number of students from each Emirate) random sampling (not detailed) Sample #: 566 included
	Al Mukhtar (2000) [19]	Overweight: BMI 25–29.0 kg/m^2^ Obesity: BMI ≥30 kg/m^2^	Cross-sectional Period: NR	Emirate: Abu Dhabi (Al Ain) Nationality: NR Gender: female Age: mean: 19.8 (1.5); groups: <20 years: 38.5%; ≥20 years: 61.5% Student residing in hostels related to UAE University	Unclear Sample #: 200
	Badr and El-Sabban (2008) [28]	Overweight: BMI 25–29.9 kg/m^2^ Obesity: BMI ≥30 kg/m^2^	Cross-sectional Period: 1996-1997	Emirate: Abu Dhabi (Al Ain) Nationality: Emirati Gender: female: 63.3%; male: 36.7% Mean age: 20.4 (1.6) (female: 20.0 (1.6); male: 21.03 (1.5)) Students in UAE University	Random sampling (not detailed) Sample #: 98
	Musaiger et al. (2003) [29]	Obesity: BMI ≥25 kg/m^2^	Cross-sectional Period: NR	Emirate: Abu Dhabi (Al Ain) Nationality: NR Gender: male Age range: 18–24 years Student residing in hostels related to UAE University	Two-stage random sampling: stage 1: hostels (simple random); stage 2: students (systematic random) Sample #: 300
	Sheikh-Ismail et al. (2009) [30]	Overweight: BMI 25–29.9 kg/m^2^ Obesity: BMI ≥30 kg/m^2^	Cross-sectional Period: October 1999–April 2000	Emirate: Abu Dhabi (Al Ain) Nationality: Emirati from all emirates Gender: female Age: 20–<30: 44.2%; 30–<60: 49.2%; >60: 6.6% Students in UAE University	For students: stratified proportionately to emirate size and conveniently from university facilities, cafeteria, student hostels, sports center, library, and classes For their family members: random (not detailed) Sample #: 724
	Kerkadi (2003) [31]	Overweight: 25 < BMI > 29.9 kg/m^2^ Obesity: BMI ≥30 kg/m^2^ (WHO classification) Hypertension (NR) Diabetes (NR)	Cross-sectional Period: NR	Emirate: Abu Dhabi (Al Ain) Nationality: NR Age range: 18–25 years Students in UAE University	Convenient sampling Sample #: 400
	Musaiger and Radwan (1995) [32]	Overweight: BMI 25–29.9 kg/m^2^ Obesity: BMI 30+	Cross-sectional Period: 1993	Emirate: Abu Dhabi (Al Ain) Nationality: Emirati: 91.6%; others: 8.4% Gender: female Mean age: 19.7 (1.3) (range: 18–30 years) Students in UAE University	Convenient sampling Sample #: 215
	Papandreou et al. (2015) [33]	Overweight (not defined) Obesity (not defined)	Cross-sectional Period: 2014	Emirate: NR Nationality: NR Gender: female Mean age: 20.55 (2.25) Students in 1 public university	Convenient sampling Sample #: 243

Obesity in community	Ng et al. (2011) [3]	Adults: (1) Overweight: ≥25 BMI <30 kg/m^2^ (2) Obesity: BMI ≥30 kg/m^2^ (WHO, 2000) Children and adolescents (<19 years): IOTF cutoffs	Cross-sectional Period: 2009-2010	Emirate: All (national) Nationality: Emirati Adult women: ≥19 years; adolescents: 11–18 years; children: 6–10 years	Multistage random sampling: stage 1: census enumeration area in the urban areas or a village in the rural areas; stage 2: households (randomization method not detailed); participants (not detailed) Sample #: households: 628 (adult women: 478; adolescents: women: 143 and men: 133; children: women: 126 and men: 127)
	Carter et al. (2004) [34]	Overweight: BMI 25–29.9 kg/m^2^ Obesity: BMI ≥30 kg/m^2^ (NHLBI)	Cross-sectional Period: September 2000–August 2001	Emirate: Abu Dhabi (Al Ain) Nationality: Emirati citizen (by birth: 79%; by marriage: 21%) Gender: female Mean age: 34.3 (14.7) Community-dwelling	Stratified multistage random sampling: stage 1: living areas (randomization unclear); stage 2: houses (systematic randomization); stage 3: all women living in chosen houses Sample #: 535

Metabolic syndrome	Mehairi et al. (2013) [35]	Metabolic syndrome (IDF definition) WC ≥90th percentile or ≥94th percentile; cut points for youth aged ≥16, TG ≥150 mg/dL (1.7 mmol/L), HDL-C <40 mg/dL (1.03 mmol/L) or <50 mg/dL (1.29 mmol/L) for female adolescents aged ≥16, FBG >100 mg/dL (5.6 mmol/L), and BP ≥130/80 mmHg	Cross-sectional Period: March–April 2010	Emirate: Abu Dhabi (Al Ain) Nationality: Emirati: 52%; others: 48% Gender: male: 51.6%; female: 48.4% Mean age: 15.4 (1.8) (range 12–18 years) Schoolchildren (public and private)	Two-stage PPS random sampling: stage 1: schools (randomly selected by using SPSS Software); stage 2: students sampled proportional to the enrollment size of each school (self-weighting) Sample #: 1,018
	Al Dhaheri et al. (2016) [36]	Metabolic syndrome ≥3 of the following: (1) elevated WC (≥80 cm); (2) hypertriglyceridemia (TG ≥150 mg/dL or drug treatment for elevated TG); (3) reduced HDL-C (<50 mg/dL or drug treatment for reduced HDL-C); (4) elevated BP (SBP >130 mmHg and/or DBP >85 mmHg or use of antihypertensive drugs); (5) elevated FBG (≥100 mg/dL or use of hypoglycemic medication) (IDF and AHA/NHLBI)	Cross-sectional Period: 2013-2014	Emirate: Abu Dhabi (Al Ain) Nationality: Emirati Gender: Female Mean age: 20.4 (1.7) (range 17–25 years) Students in UAE University	Stratified random sampling: stratification by college, followed by random subsample of 10% of each college (unclear randomization) Sample #: 555
	Malik and Razig (2008) [17]	Metabolic syndrome: NCEP and IDF definition (ethnicity-specific cutoff levels of WC to define central obesity ≥90 cm for South Asian men and ≥94 cm for men from other nationalities; for women, irrespective of ethnicity: ≥ 80 cm; high WHR: ≥0.95 for men and ≥0.90 for women)	Cross-sectional Period: October 1999–June 2000	Emirate: All (national) Nationality: Emirati: 42%; others: 58% Gender: male: 41.3%; female: 58.7% Mean age: 41.45 (11.7)	Participants recruited from the 2000 Emirates National Diabetes study and screening for risk factors for Coronary Artery Disease Study Multistage, stratified, cluster random sampling (not detailed) Sample #: 4,097

Dyslipidemia	Agarwal et al. (1995) [37]	Dyslipidemia (elevated total cholesterol) Borderline high: 200–239 mg/dL High: 240 mg/dL (NCEP guidelines)	Cross-sectional Period: NR	Emirate: NR Nationality: UAE nationals: 26.6%; Arabs (non-UAE): 45.9%; non-Arabs: 27.5% Gender: female: 24.6%; male: 75.4% Age: <51 years: 85.1%; >51 years: 14.9%	Convenient sampling (recruitment from urban public sites, e.g., shopping malls, mosques, etc.) Sample #: 834

Hypertension	Abdulle et al. (2014) [38]	Prehypertension: BP ≥90th and <95th CDC percentiles for age and sex Hypertension: BP ≥95th CDC percentile for age and sex	Cross-sectional Period: January 2011–December 2011	Emirate: Abu Dhabi Nationality: Emirati Gender: female: 47.3%; male: 52.7% Mean age: female: 11.0 (3.4); male: 11.7 (3.5) (range 6–17 years) Schoolchildren (public)	Two-stage random sampling: stage 1: public schools (stratified to collect a similar number of boys and girls); stage 2: students (proportional to school size) Sample #: 999 (405 non-Emirati and 36 Emirati adults were excluded)
	El Shahat et al. (1999) [39]	Hypertension: SBP >140 mmHg and/or DBP >90 mmHg, and/or self-reported treatment with antihypertensive medications (JNC-VI on detection, evaluation, and treatment of high blood pressure)	Cross-sectional Period: 1997	Emirate: Sharjah Nationality: Emirati Gender: female: 53%; male: 47% Age: 17–30: 26%; 31–50: 46%; >50: 28% (range: 18–75 years)	Stratified (unclear) systematic random sampling (PHC) and census of governmental departments' employees Sample #: 3,150

Diabetes	El Mugamer et al. (1995) [40]	Diabetes: random BG (taken 2–4 hours after a meal) ≥11.1 mmol/L (WHO) Hypertension: SBP >140 mmHg and/or DBP >90 mmHg Obesity: BMI ≥30 kg/m^2^	Cross-sectional Period: 1989-1990	Emirate: Abu Dhabi (Al Ain) (Zakher (urban) and Al Hayer and Wagan (rural) areas) Nationality: Emirati Gender: female: 61.8%; male: 38.2% Age: >19 years	Purposive for the locations (to increase the Bedouin-derived population); unclear for participants Sample #: 322
	Saadi et al. (2007) [41]	Prediabetes: impaired fasting glucose (venous blood glucose: 5.6–6.9 mmol/L) or impaired glucose tolerance (2 h post-OGTT venous blood glucose: 7.8–11.0 mmol/L) Diabetes: fasting venous blood glucose concentration ≥7.0 mmol/L and/or 2 h post-OGTT venous blood glucose concentration ≥11.1 mmol/L (WHO expert group)	Cross-sectional Period: December 2005–November 2006	Emirate: Abu Dhabi (Al Ain) Nationality: Emirati Gender: female: 50.9%; male: 49.1% Age: ≥18 years	Two-stage sample: stage 1: houses (simple random sample of houses listed in the electricity department); stage 2: all men and nonpregnant women living in chosen houses Sample #: 452 houses (2455 adults, including 2396 for whom diabetes status was available)

Multiple risk factors	Malik et al. (2005) [18]	Abnormal glucose tolerance: WHO expert group recommendation Diabetes: FBG ≥7.0 mmol/L and/or 2 h BG ≥11.1 mmol/L Impaired fasting glycemia: FBG: 6.1–6.9 mmol/L IGT: 2 h venous BG: 7.8–11.0 mmol/L on the OGTT Hypertension: SBP ≥140 mmHg and/or DBP ≥90 mmHg Central obesity: WHR ≥0.95 for men and ≥0.90 for women Preobesity: BMI 25–29.9 kg/m^2^ Obesity: BMI ≥30 kg/m^2^	Cross-sectional Period: 1999-2000	Emirate: All (national) Nationality: Emirati: 40%; others: 60% Gender: male: 43%; female: 57% Age: ≥20 years	Participants recruited from the 2000 Emirates National Diabetes study and screening for risk factors for Coronary Artery Disease Study Multistage, stratified, cluster random sampling (not detailed) Sample #: 5,844
	Yusufali et al. (2015) [42]	Dyslipidemia: history of known or treated dyslipidemia (receiving cholesterol-lowering medication) or total cholesterol ≥200 mg/dl or HDL-C <40 mg/dl Hypertension: history of known and treated hypertension (receiving antihypertensive medication) or SBP ≥140 mm Hg or DBP ≥90 mm Hg Obesity: BMI ≥30.0 kg/m^2^ Diabetes: history of known and treated diabetes (receiving antihyperglycemic medication) or HbA1c ≥6.5% Central obesity: WC ≥102 cm in male and ≥88 cm in female	Cross-sectional Period: September-October 2012	Emirate: Dubai, Abu Dhabi, Sharjah, Fujairah, and Ras Al Khaimah Nationality: Emirati: 6.7%; other Arabs: 9.9%; South Asians: 73.7%; other Asians: 4.6%; others: 5.1% Mean age: 38 (11) Gender: male: 75%; female: 25%	Opportunistic sampling (convenient recruitment from shopping malls, outpatient health care facilities, and labor camps) Sample #: 4,128
	Baynouna et al. (2008) [43]	Diabetes: FBG >125 mg/dL, use of diabetes medications, or self-reported diabetes (ADA) Prehypertension: BP ≥120/80 mm Hg on more than 2 occasions Hypertension: BP>140/90 mm Hg on both visits (JNC criteria) Obesity: BMI ≥30 kg/m^2^ Metabolic syndrome: ≥3 of the following: central obesity, high TG, low HDL-C, high BP, or IFG (ATP III criteria) Central obesity (not defined) Dyslipidemia (not defined)	Cross-sectional Period: February 2004–February 2005	Emirate: Abu Dhabi Nationality: Emirati Gender: female: 51.8%; male: 48.2% Mean age: 44.1 (range: 25–68)	Two-stage sampling: stage 1: selection of PHCs stratified by geography (choose the busiest if more than 1); stage 2: random selection from lists of possession of a health card, stratified by gender (randomization method not detailed) Sample #: 817
	Hajat and Harrison (2010) [44]	Overweight (not defined) Obesity (not defined) Central obesity: elevated WC with ethnicity-specific values Prediabetes: HbA1c 5.7%–6.4% (ADA classification) Diabetes: HbA1c ≥6.5% or random glucose >11.1 mmol/L or self-reported history of diabetes warranting treatment Framingham Risk Score	Cross-sectional Period: April 2008–April 2010	Emirate: Abu Dhabi Nationality: Emirati Mean age: 35.2 (13.8)	All individuals included in the WEQAYA screening program Sample #: 173,501
	Hajat et al. (2012) [20]	Overweight: BMI 25 to 29.9 kg/m^2^ Obesity: BMI ≥30 kg/m^2^ Central obesity: WHR ≥0.85 for women and ≥0.9 for men Hypertension: self-reported past history of high BP requiring medication or a single elevated clinical BP reading (SBP ≥140 mmHg or DBP ≥90 mmHg) Dyslipidemia: self-reported past history of abnormal cholesterol levels requiring medication or a measured LDL-C ≥4.1 mmol/L or HDL-C ≤1.0 mmol/L	Cross-sectional Period: April 2009–June 2010	Emirate: Abu Dhabi Nationality: Emirati Gender: female: 57%; male: 43% Mean age: 36.82 (14.3)	All individuals included in the WEQAYA screening program Sample #: 50,138

Employees	Hossain and Malik (1998) [45]	IGT: FBG <7.8 mmol/L and 2-hour BG: 7.8–11.1 mmol/L Diabetes: FBG >7.8 mmol/L or 2-hour BG >11.1 mmol/L Elevated blood cholesterol: fasting total cholesterol >200 mg/dl Obesity: BMI ≥24.99 kg/m^2^ and WHR >1.0	Cross-sectional Period: May 1995–January 1996	Emirate: Abu Dhabi Gender: male Age range: 35–49 years Other characteristics: office based in a group of petroleum companies	Convenient sampling Sample #: 358
	Newson-Smith (2010) [46]	Obesity: ≥30 kg/m^2^ Diabetes (not defined) Hypertension (not defined)	Cross-sectional Period: 2005/2008	Nationality: Emirati: 13.3%; Indians: 43.1%; Egyptians: 15.1%; Filipinos: 7.3%; others: 21.2% Gender: male Mean age: 37.3 (range: 19–64 years) Other characteristics: oil and gas company workers	Unclear Sample #: 1,037
Incidence	Sreedharan et al. (2015) [13]	Diabetes: FBG ≥126 mg/dL or previous first diagnosis of diabetes or documented diabetes by a physician Impaired fasting glucose: FBG 110–126 mg/dL or documented impaired fasting glucose by a physician	Retrospective cohort Period: January 2010–December 2010	Emirati and non-Emirati in Ajman	Exhaustive (all cases treated in 5 PHCs and 2 general hospitals where most of the diabetic patients are presumed to be managed) Sample #: NR

BMI: body mass index; NHANES: National Health and Nutrition Examination Survey; PPS: probability proportional to size; IOTF: International Obesity Task Force; SD: standard deviation; WHO: World Health Organization; CDC: Centers for Disease Control and Prevention; NR: not reported; UAE: United Arab Emirates; NHLBI: National Heart, Lung, and Blood Institute; IDF: International Diabetes Federation; WC: waist circumference; HDL-C: high-density lipoprotein cholesterol; FBG: fasting blood glucose; BP: blood pressure; TG: triglycerides; SBP: systolic blood pressure; DBP: diastolic blood pressure; AHA: American Heart Association; NCEP: National Cholesterol Education Program; WHR: waist-to-hip ratio; JNC: Joint National Committee; OGTT: oral glucose tolerance test; HbA1c: hemoglobin A1c; ADA: American Diabetes Association; ATP: Adult Treatment Panel; LDL-C: low-density lipoprotein cholesterol; BG: blood glucose; PHC: primary health care center.

**Table 2 tab2:** Epidemiology of cardiometabolic diseases in the United Arab Emirates and risk of bias in the included studies.

	Author and year	Prevalence of cardiometabolic disease(s)	Risk factors (multivariate analysis)
Obesity in children	Al Hourani et al. (2003) [21]	At risk for overweight: 14% Overweight: 9% (at risk for overweight and obesity: Abu Dhabi: 21%; Sharjah: 24%; Dubai: 27%; Ras Al Khaimah: 22%; Fujairah: 30%)	Not assessed
	Al-Haddad et al. (2005) [22]	Overweight: 18.6% (calculated) (boys: 17.1%; girls: 20.1%) Obesity: 7.4% (calculated) (boys: 7.7%; girls: 7.1%)	Not assessed
	Malik and Bakir (2007) [15]	Overweight: girls: 19.8%, 95% CI: 18.3–21.6; boys: 19.2%, 95% CI: 17.6–20.9 Obesity: girls: 12.4%, 95% CI: 11.1–13.8; boys: 13.1%, 95% CI: 11.6–14.5 Emirati: Overweight: girls: 17.6%, 95% CI: 15–20.2; boys: 17.3%, 95% CI: 15.3–19.9 Obesity: girls: 14.3%, 95% CI: 11.5–16.7; boys: 12.7%, 95% CI: 10.4–14.6 Others: Overweight: girls: 25%, 95% CI: 22.8–27.5; boys: 20.5%, 95% CI: 18.6–22.5 Obesity: girls: 13.5%, 95% CI: 11.8–15.4; boys: 14.6%, 95% CI: 12.5–16.7	Obesity: Predictors: Non-Emirati boys (OR = 1.783, CI 1.499–2.121) Non-Emirati girls (OR = 1.767, CI 1.48–2.102) Birth outside the UAE (OR = 1.173, CI 1.015–1.306) Girls living in rural areas (OR = 1.614, CI 1.348–2.001) Protective factors: Boys living in rural areas (OR = 0.732, CI 0.591–0.912) Overweight: Protective factors: Non-Emirati boys (OR = 0.662, CI 0.572–0.769) Non-Emirati girls (OR = 0.569, CI 0.461–0.703) Boys living in rural areas (OR = 0.785, CI 0.629–0.974)
	Abdulrazzaq et al. (2011) [16]	Under 5 years: Overweight: boys: 13.45%; girls: 12.98% Obesity: boys: 6.06%; girls: 6.54% At age 13–17: Overweight: boys: 14.16%; girls: 15.16% Obesity: boys: 6.08%; girls: 9.94% At age 18: Overweight: boys: 25%; girls: 30% Obesity: boys: 7%; girls: 10%	Not assessed
	Al-Haddad et al. (2000) [14]	Overweight: 9.0% (boys: 8.5%; girls: 9.3%) Obesity: 7.9% (boys: 7.9%; girls: 7.9%)	Not assessed
	Al Blooshi et al. (2016) [23]	Emirati: Overweight: CDC: 17%; IOTF: 16%; WHO: 4.5% Obesity: CDC: 20%; IOTF: 14%; WHO: 30.3% Residents: Overweight: CDC: 14% (calculated figure) Obesity: CDC: 18% (calculated figure)	Age and gender: prevalence of overweight, obesity, and extreme obesity increased linearly with age in children 3–12 y (3.89% per year, *R* ^2^ ≥ 0.962); a rate 28% higher in boys than in girls
	Bin Zaal et al. (2009) [24]	Overweight: girls: 13.1%; boys: 18.5% Obesity: girls: 20.5%; boys: 22.2%	Protective factors: Girls: always eating breakfast (OR = 0.5; 95% CI 0.2–1.0); eating breakfast at school (OR = 3.4; 95% CI 1.6–7.4); frequently snacking in between breakfast and lunch (OR = 0.5; 95% CI 0.3–0.9); meat consumption ≥4 times/week (OR = 0.1; 95% CI 0.02–1.0); chocolates and sweets consumption ≥4 times/week (OR = 0.5; 95% CI 0.3–0.8); soft drinks consumption ≥4 times/week (OR = 0.5; 95% CI 0.3–0.8); fast foods consumption ≥4 times/week (OR = 0.5; 95% CI 0.3–0.8) Boys: fruit consumption: ≥4 times/week (OR = 0.6; 95% CI 0.4–1.0) Predictors: Boys: eating breakfast at school (OR = 3.0; 95% CI 1.1–8.3)
	Al Junaibi et al. (2013) [25]	Overweight: 14.7% (boys: 11.7%; girls: 17.6%) Obesity: 18.9% (boys: 20.7%; girls: 17.0%) Emirati nationals: Overweight: 14.2% (boys: 11.6%; girls: 16.7%) Obesity: 19.8% (boys: 21.4%; girls: 18.1%)	Positive correlation between child's BMI percentiles and parental BMI (for every kg/m^2^ of parental BMI, the child's BMI percentile increased by 2.34 percentile points) Negative correlation between child's BMI percentiles and dairy consumption (each additional daily dairy consumption was associated with a reduction in BMI by 2.52 percentile points; *p* < 0.001)
	Musaiger et al. (2012) [26]	IOTF: Overweight: 15.24% (boys: 16.8%; girls: 13.6%) Obesity: 13.07% (boys: 19.1%; girls: 6.6%) CDC: Overweight: boys: 11.1%; girls: 11.5% Obesity: boys: 22.5%; girls: 7%	Not assessed

Obesity in university students	Amine and Samy (1996) [27]	Overweight: 10.8%; obesity: 30.6% Abu Dhabi: overweight: 12.3%; obesity: 31.6% Dubai: overweight: 4%; obesity: 31.3% Sharjah: overweight: 10.1%; obesity: 28.4% Ras Al Khaimah: overweight: 15.2%; obesity: 29.1% Others: overweight: 11.8%; obesity: 31.4%	Significant association (bivariate analysis) between obesity and: Obesity during childhood Obesity among parents (both or mother or father only) Eating between meals (regularly or occasionally)
	Al Mukhtar (2000) [19]	Overweight: 24.0% (<20 years: 27.3%; ≥20 years: 21.9%) Obese: 7.5% (<20 years: 6.5%; ≥20 years: 8.2%)	Not assessed
	Badr and El-Sabban (2008) [28]	Overweight and obesity: 13.3% Female: overweight and obesity: 8.1% Male: overweight and obesity: 22.2%	Positive correlation between BMI of males and their fathers' BMI (*r* = 0.51; *p* < 0.0001)
	Musaiger et al. (2003) [29]	Obesity: 35.7%	Predictors: Family history of obesity (RR = 1.88); not practicing sport (RR = 1.77)
	Sheikh-Ismail et al. (2009) [30]	Overweight: 27% (age group: 20–<30: 21%; 30–<60: 33%; >60: 15%) Obesity: 16% (age group: 20–<30: 8%; 30–<60: 24%; >60: 14%)	Not assessed
	Kerkadi (2003) [31]	Obesity: 6.7% Overweight: 19.4% Reported hypertension: 2.8% Reported diabetes: 1.4%	Significant association in bivariate analysis between obesity and higher consumption of cereals and fruits (*p* < 0.005)
	Musaiger and Radwan (1995) [32]	Overweight: 19% Obesity: 9.8%	No statistical significance was found for any of the assessed risk factors
	Papandreou et al. (2015) [33]	Overweight and obesity: 28.4%	Not assessed

Obesity in community	Ng et al. (2011) [3]	Adult female: overweight: 31.4%; obesity: 34.2%; elevated WC: 53.2% Adolescent female: overweight: 20.5%; obesity: 19.7% Adolescent male: overweight: 16.2%; obesity: 11.7% Children (girls): overweight: 23.6%; obesity: 17.1% Children (boys): overweight: 9.1%; obesity: 15.9%	Not assessed
	Carter et al. (2004) [34]	Overweight: 27% Obesity: 35%	Age (OR = 1.05; 95% CI 1.04–1.07)

Metabolic syndrome	Mehairi et al. (2013) [35]	Metabolic syndrome: 13% (boys: 22%; girls: 4%) Elevated WC: boys: 22%; girls: 4% IFG: boys: 13%; girls: 6% Low HDL: boys: 88%; girls: 74% Elevated TG: boys: 5%; girls: 1% Hypertension: boys: 5%; girls: 8% Overweight or obesity: 34.6% (boys: 39%; girls: 30%)	Predictors of metabolic syndrome: Boys: screen time (aOR: 1.08, 95% CI: 1–1.17); BMI (aOR: 1.26, 95% CI: 1.2–1.33) Girls: BMI (aOR: 1.22, 95% CI: 1.2–1.33)
	Al Dhaheri et al. (2016) [36]	Metabolic syndrome: 6.8% (95% CI: 5–9%) (reduced HDL-C: 48.8%; elevated WC: 18.2%; elevated FBG: 9.7%; elevated BP: 5.4%; hypertriglyceridemia: 1.4%) (At least 1 component: 38.4%; 2 components: 11.2%; 3 components: 4.9%; 4 components: 1.8%; 5 components: 0.2%)	Overweight (aOR = 3.8, 95% CI: 1.15–12.52) Obesity (aOR = 11.2, 95% CI: 3.1–40.9) WHR >0.8 (aOR = 3.04, 95% CI: 1.10–8.44) HbA1c 5.6–6.4% (aOR = 8.92; 95% CI: 3.39–23.48) HbA1c>6.5% (aOR = 22.5, 95% CI: 6.37–79.42)
	Malik and Razig (2008) [17]	Metabolic syndrome: NCEP definition: 38.4% (36.9–39.9) (male: 32.4% (30–34.3); female: 47.0% (44.6–49.3)) IDF definition: 40.5% (39–42) (male: 32.9% (30.7–35.2); female: 45.9% (43.9–47.9)) Abdominal obesity: NCEP definition: 44.8% (43.3–46.3) (male: 25.3% (23.3–27.4); female: 60% (58–62)) IDF definition: 69.9% (68.5–71.3) (male: 63.4% (61.1–65.7); female: 74.6% (72.7–76.2)) High BP: 38.4% (36.9–39.9) (male: 47% (44.6–49.3); female: 32.4% (30–34.3)) High fasting plasma glucose: 50.7% (male: 46.4% (44.1–48.8); female: 53.7% (51.7–55.6)) High plasma TG: 33.5% (32.1–34.9) (male: 43.6% (41.2–46); female: 26.4% (24.7–28.2)) Low HDL-C: 53.9% (52.3–55.4) (male: 43.4% (41.1–45.8); female: 61.2% (59.3–63.1)) Emirati: Metabolic syndrome: NCEP definition: 42.9% (40.1–44.8) (male: 31% (27.1–38.9); female: 51.2% (48.1–54.1)) IDF definition: 41.8% (39.5–44.1) (male: 37.1% (33–41.5); female: 44.3% (41.5–47.1))	Predictors of metabolic syndrome for both definitions: Increasing age (≥40 years), female gender, and family history of diabetes

Dyslipidemia	Agarwal et al. (1995) [37]	Dyslipidemia (total cholesterol) UAE nationals: borderline: 33.3%; high: 19.8% Arabs (non-UAE): borderline: 29.5%; high: 19.6% Non-Arabs: borderline: 31%; high: 11.4% Female: borderline: 34.2%; high: 15.1% Male: borderline: 29.9%; high: 18.1% <51 years: borderline: 36.3%; high: 16.5% >51 years: borderline: 34.7%; high: 22.6%	Not assessed

Hypertension	Abdulle et al. (2014) [38]	Prehypertension: 10.9% (male: 10.5%; female: 11.4%) Hypertension: 16.5% (male: 15.4%; female: 17.8%) Systolic hypertension: 14.6% (male: 14.4%; female: 14.8%) Diastolic hypertension: 4.9% (male: 2.5%; female: 7.4%)	Predictors of systolic BP *Z*-scores: Age (*B*(SE) = –0.010 (0.005)); BMI CDC percentile (*B*(SE) = 0.006 (0.001)) Predictors of diastolic BP *Z*-scores: BMI CDC percentile (*B*(SE) = 0.002 (0.0003)); sex (*B*(SE) = –0.113 (0.025)) Positive relationship between BP *Z*-scores and weight status in all age groups and both sexes
	El-Shahat et al. (1999) [39]	Hypertension: 36.6% (calculated according to census in Sharjah: 31.6%) Stage I: 32%; stage II: 4%; stage III: 0.05% Female: 33.7% (stage I: 28.3%; stage II: 4.8%; stage III: 0.05%) Male: 40.3% (stage I: 36.7%; stage II: 2.9%; stage III: 0.6%)	Not assessed

Diabetes	El Mugamer et al. (1995) [40]	(Age-adjusted prevalence) Diabetes: 6% (male: 5.8%; female: 6.1%; Shamsi: 18.7%; others: 4.2%; rural: 4.0%; urban: 9.2%) Obesity: male: 10.7%; female: 27.4%; Shamsi: 16.1%; others: 21.8%; rural: 17.9%; urban: 26.7% Elevated SBP: male: 23.0%; female: 19.7%; Shamsi: 12.9%; others: 22.2%; rural: 19.2%; urban: 24.9% Elevated DBP: male: 17.9%; female: 12.8%; Shamsi: 16.1%; others: 14.7%; rural: 12.1%; urban: 18.3%	Predictors of higher FBG: Age 40–59: *B* = 1.59, SE = 0.4; ≥60 y: *B* = 1.01, SE = 0.43; tribe (Shamsi): *B* = 2.07, SE = 0.60; residence (urban): *B* = 1.56, SE = 0.37 Predictors of higher BMI: Age 40–59: *B* = 1.85, SE = 0.62; gender (female): *B* = 1.93, SE = 0.54; tribe (Shamsi): *B* = −2.18, SE = 0.94; residence (urban): *B* = 1.82, SE = 0.59 Predictors of higher SBP: Age 40–59: *B* = 11.5, SE = 2.7; ≥60 y: *B* = 21.2, SE = 2.9 Predictors of higher DBP: Age 40–59: *B* = 7.9, SE = 1.5; ≥60 y: *B* = 6.5, SE = 1.7; gender (female): *B* = −3.0, SE = 1.3
	Saadi et al. (2007) [41]	Reported: Diabetes: 10.2% (male: 9.4%; female: 11.1%) Segi-standardized rates among 30- to 64-year-olds: 20.6% (male: 17.7%; female: 22.1%) Measured: Diabetes: 25.9% (diagnosed: 15.3%; undiagnosed: 10.7%); male: 27.1% (diagnosed: 18.9%; undiagnosed: 8.2%); female: 25.5% (diagnosed: 13.5%; undiagnosed: 12.0%) Prediabetes: 22.8% (male: 19.7%; female: 24.3%) Adjusted for the probability of inclusion in the study: Diabetes: 17.1% (diagnosed: 10.5%; undiagnosed: 6.6%) Prediabetes: 20.2% Age-standardized rates among 30- to 64-year-olds: Diabetes: 29.0% (diagnosed: 15.0%; undiagnosed: 14.0%) Prediabetes: 24.2%	Predictors of undiagnosed diabetes: BMI: *B* = 0.088; age: *B* = 0.059

Multiple risk factors	Malik et al. (2005) [18]	Diabetes: Crude: 20.2% (male: 21.5% (including newly diagnosed: 35.6%); female: 19.2% (including newly diagnosed: 44.9%)) Age-standardized rates (95% CI): 21.4% (20.4–22.4) (male: 20.4% (18.8–22); female: 22.3% (20.9–23.7)) IFG: Crude: 6.5% (male: 4.5%; female: 8%) Age-standardized rates (95% CI): 6.6% (6–7.2) (male: 4.5% (3.7–5.3); female: 7.2% (6.3–8.1)) Overweight: crude: 40% Obesity: crude: 33% Emirati: Diabetes: crude: 24.5%; age-adjusted rate: 25.1% IFG: crude: 8.8%; age-adjusted rate: 8.5% Obesity: crude: 37%	Predictors of diabetes: WHR: OR = 1.73, 95% CI: 1.18–2.55; age: OR = 1.06, 95% CI: 1.05–1.07; SBP: OR = 1.01, 95% CI: 1.001–1.01; BMI: OR = 1.04, 95% CI: 1.02–1.05 Protective factors for diabetes: Nationality: Shwam: OR = 0.45, 95% CI: 0.36–0.57; Egypt/North Africa: OR = 0.7, 95% CI: 0.55–0.9; Sudan/East Africa: OR = 0.6, 95% CI: 0.45–0.8
	Yusufali et al. (2015) [42]	Mean 10-year Framingham CVD Risk Score: 5.3 (7.1) (male: 5.5 (7.3); female: 4.7 (6.0)) (Emirati: 7.2) Diabetes: 31.6% (Emirati: 46.2%; other Arabs: 29.3%; South Asians: 31.7%; other Asians: 22.1%; others: 23.2%) Hypertension: 30.6% (Emirati: 30.2%; other Arabs: 22.9%; South Asians: 31.5%; other Asians: 39%; others: 24.2%) Dyslipidemia: 68.5% (Emirati: 666.2%; other Arabs: 69.3%; South Asians: 71%; other Asians: 53.2%; others: 47.9%) Overweight: 41.9% (Emirati: 34.3%; other Arabs: 41.9%; South Asians: 43.1%; other Asians: 40.4%; others: 35.8%) Obesity: 19.6% (Emirati: 46.6%; other Arabs: 40.9%; South Asians: 14.1%; other Asians: 16%; others: 25.5%) Central obesity: 24% (Emirati: 55.8%; other Arabs: 43.5%; South Asians: 18.5%; other Asians: 27.9%; others: 33%)	Predictors of risk factors: Male gender (OR: 3.441; 95% CI: 2.930 to 4.042) Protective factor: Age (per 10 years) (OR: 0.834; 95% CI: 0.784 to 0.886)
	Baynouna et al. (2008) [43]	Diabetes: 23.3% (self-reported: 19.5%; additionally measured: 3.8%) (female: 18.4%; male: 10.4%) Prehypertension: 7.3% Hypertension: 20.8% (self-reported: 20%; additionally measured: 0.8%) (female: 19.8%; male: 21.8%); Obesity: 37.3% (female: 46.5%; male: 28.3%) Central obesity: 39% (female: 59.9%; male: 37.2%) Metabolic syndrome: 22.7% (female: 24.2%; male: 21.3%) Dyslipidemia: 58.9% (female: 53.9%; male: 64%)	Not assessed
	Hajat and Harrison (2010) [44]	Overweight: 32% Obesity: 35% Central obesity: 57% Either prediabetes or diabetes: 44% Framingham CVD Risk Score: >10%: male: 20.32%; female: 9.51%; overall: 14.22% >20%: male: 7.59%; female: 2.56%; overall: 4.75%	Not assessed
	Hajat et al. (2012) [20]	Obesity: crude: 35.4% (female: 38.3%; male: 31.6%); ASR: 41.1% (40.7; 41.5) Morbid obesity: 5% (female: 6%; male: 4%) Overweight: crude: 31.9% (female: 28.8%; male: 36.1%); ASR: 34% (33.6; 34.4) Central obesity: crude: 54.8% (female: 51.9%; male: 58.6%); ASR: 62.4% (61.9; 62.8) Dyslipidemia: crude: 44.2% (female: 33.9%; male: 57.7%); ASR: 50.7% (50.3; 51.2) Hypertension: crude: 23.1% (female: 20.9%; male: 26%); ASR: 29.2% (28.8; 29.6) Prediabetes: crude: 27.1% (female: 26.5%; male: 27.8%); ASR: 29.5% (29.1; 29.9) Diabetes: crude: 17.6% (female: 17.9%; male: 17.4%); ASR: 24.6% (24.2; 25) (newly diagnosed: 35%; self-reported history: 65%)	Not assessed

Workers	Hossain and Malik (1998) [45]	IGT: 18% Diabetes: 10% Obesity (defined by BMI): 68%, and obesity (defined by WHR): 31% Elevated blood cholesterol: 74%	Predictors of obesity (elevated BMI): Peninsular Arab: aOR: 3.06 (1.44–6.54); Shwam: aOR: 4.14 (1.96–8.75); Egyptian: aOR: 4.64 (1.4–15.21); WHR >1: aOR: 3.31 (1.77–6.18) Predictor of obesity (elevated WHR): BMI >25: aOR: 3.57 (1.89–6.73) Predictor of elevated total cholesterol: age 45–49: aOR: 2.8 (1.47–5.32) Protective factors of abnormal glucose tolerance: Shwam: aOR: 0.37 (0.17–0.78); Egyptian: aOR: 0.31 (0.1–0.96) Protective factors of obesity (elevated WHR): Afro-Arab: aOR: 0.14 (0.03–0.64); European: aOR: 0.15 (0.03–0.72); medium physical activity: aOR: 0.4 (0.19–0.83); heavy physical activity: aOR: 0.27 (0.09–0.8)
	Newson-Smith (2010) [46]	Preemployment assessment: Obesity: 16.6% (Emiratis: 31.9%; Pakistanis: 16.6%; Filipinos: 5.9%; Indians: 2.4%) Diabetes: 2.5% (Emiratis: 3.6%; Pakistanis: 0%; Filipinos: 2.9%; Indians: 1.6%) Hypertension: 15.5% (Emiratis: 0%; Pakistanis: 16%; Filipinos: 32.4%; Indians: 28.4%) At periodic health evaluation: Obesity: 8.6% (Emiratis: 29%; Pakistanis: 43%; Filipinos: 8%; Indians: 7%) Diabetes: 11.3% (Emiratis: 0%; Pakistanis: 21.4%; Filipinos: 9.5%; Indians: 11.6%) Hypertension: 37% (Emiratis: 12.5%; Pakistanis: 50%; Filipinos: 47.6%; Indians: 37.3%)	Not assessed

Incidence	Sreedharan et al. (2015) [13]	Overall incidence in ≥20 years: 4.8/1,000 PY Gender-specific incidence rate: male: 3.3/1,000 PY; female: 6.3/1,000 PY Highest incidence rate for both genders: age group: 55–59 (male: 23.4/1,000 PY; female: 32.4/1,000 PY)	ASR in male and female was almost similar until the age of 39 years; then, females ≥40 years showed a higher incidence rate than males

CI: confidence interval; OR: odds ratio; UAE: United Arab Emirates; CDC: Centers for Disease Control and Prevention; IOTF: International Obesity Task Force; WHO: World Health Organization; BMI: body mass index; aOR: adjusted odds ratio; HDL-C: high-density lipoprotein cholesterol; WC: waist circumference; FBG: fasting blood glucose; BP: blood pressure; HbA1c: Hemoglobin A1c; NCEP: National Cholesterol Education Program; IDF: International Diabetes Federation; TG: triglycerides; SE: standard error; IFG: impaired fasting glucose; WHR: waist-to-hip ratio; CVD: cardiovascular disease; ASR: age-standardized rate; IGT: impaired glucose tolerance; PY: person-years.

**Table 3:** Risk of bias of included epidemiological studies.

Author and year	Was the study's target population a close representation of the national population in relation to relevant variables?	Was the sampling frame a true or close representation of the target population?	Was some form of random selection used to select the sample or was a census undertaken?	Was the likelihood of nonresponse bias minimal?	Were data collected directly from the subjects (opposed to a proxy)?	Was an acceptable case definition used in the study?	Had the study instrument that measured the parameter of interest been tested for reliability and validity (if necessary)?	Was the same mode of data collection used for all subjects?	Was the length of the shortest prevalence period for the parameter of interest appropriate?	Were the numerator(s) and denominator(s) for the parameter of interest appropriate?	Summary item on the overall risk of study bias
Agarwal et al. (1995) [37]	High	High	High	High	Low	Low	Low	Low	Low	Low	Medium
Al-Dhaheri et al. (2016) [36]	High	Low	Low	High	Low	Low	High	Low	Low	Low	Medium
Al Junaibi et al. (2013) [25]	High	High	High	Low	Low	Low	Low	Low	Low	Low	Medium
Al-Mukhtar et al. (2000) [19]	High	High	High	Low	Low	Low	Low	Low	Low	Low	Medium
Badr and El-Sabban (2008) [28]	High	High	High	Low	Low	Low	Low	Low	Low	Low	Medium
Hossain and Malik (1998) [45]	High	Low	High	Low	Low	Low	High	High	Low	Low	Medium
Kerkadi (2003) [31]	High	High	Low	Low	Low	Low	High	Low	Low	Low	Medium
Musaiger and Radwan (1995) [32]	High	High	Low	Low	Low	Low	High	Low	Low	Low	Medium
Musaiger et al. (2003) [29]	High	High	Low	Low	Low	Low	High	Low	Low	Low	Medium
Papandreou et al. (2015) [33]	High	High	High	Low	Low	Low	Low	Low	Low	Low	Medium
Yusufali et al. (2015) [42]	High	High	High	High	Low	Low	High	Low	Low	Low	Medium
Abdulle et al. (2014) [38]	High	Low	Low	High	Low	Low	Low	Low	Low	Low	Low
Abdulrazzaq et al. (2011) [16]	Low	Low	Low	Low	Low	Low	Low	Low	Low	Low	Low
Al Blooshi et al. (2016) [23]	High	Low	Low	Low	Low	Low	Low	Low	Low	Low	Low
Al-Haddad et al. (2000) [14]	Low	Low	High	Low	Low	Low	Low	Low	Low	Low	Low
Al-Haddad et al. (2005) [22]	Low	Low	Low	Low	Low	Low	Low	Low	Low	Low	Low
Al-Hourani et al. (2003) [21]	Low	High	High	Low	Low	Low	Low	Low	Low	Low	Low
El-Shahat et al. (1999) [39]	High	Low	Low	Low	Low	Low	Low	Low	Low	Low	Low
Amine and Samy (1996) [27]	High	Low	Low	Low	Low	Low	Low	Low	Low	Low	Low
Baynouna et al. (2008) [43]	High	Low	Low	High	Low	Low	Low	Low	Low	Low	Low
Bin Zaal et al. (2009) [24]	High	Low	Low	Low	Low	Low	Low	Low	Low	Low	Low
Carter et al. (2004) [34]	High	Low	Low	Low	Low	Low	Low	Low	Low	Low	Low
El Mugamer et al. (1995) [40]	High	Low	Low	High	Low	Low	Low	Low	Low	Low	Low
Hajat and Harrison (2010) [44]	Low	Low	Low	Low	Low	Low	Low	Low	Low	Low	Low
Hajat et al. (2012) [20]	Low	Low	Low	Low	Low	Low	High	Low	High	Low	Low
Malik et al. (2005) [18]	Low	Low	Low	Low	Low	Low	Low	Low	Low	Low	Low
Malik and Bakir (2007) [15]	Low	Low	Low	Low	Low	Low	Low	Low	Low	Low	Low
Malik and Razig (2008) [17]	Low	Low	Low	Low	Low	Low	Low	Low	Low	Low	Low
Mehairi et al. (2013) [35]	Low	Low	Low	Low	Low	Low	Low	Low	Low	Low	Low
Musaiger et al. (2012) [26]	Low	Low	Low	Low	Low	Low	Low	Low	Low	Low	Low
Newson-Smith (2010) [46]	High	Low	Low	Low	High	Low	Low	Low	Low	Low	Low
Ng et al. (2011) [3]	Low	Low	Low	Low	Low	Low	Low	Low	Low	Low	Low
Saadi et al. (2007) [41]	Low	High	Low	Low	Low	Low	Low	Low	Low	Low	Low
Sheikh-Ismail et al. (2009) [30]	High	Low	Low	Low	Low	Low	Low	Low	Low	Low	Low
Sreedharan et al. (2015) [13]	High	Low	Low	Low	Low	Low	Low	Low	Low	Low	Low

Items were categorized as having a “high risk,” “intermediate risk,” or “low risk” of bias. Unclear or poor reporting was considered as a high risk of bias. Studies were regarded as having a high overall risk of bias if they meet less than 5 criteria, moderate risk if they meet 5 to 7 criteria, and low risk if they meet 8 or more of the 10 items.

**Table 4:** Cost of illness for diabetes from Al-Maskari et al. [12].

Study design	Sample size	Data sources	Cost components and costing approach	Perspective	Time horizon	Economic burden (annual cost/patient) (US$, 2004)	Major limitations
Cross-sectional	150 (recruited from 2 outpatient clinics at Al Ain, Abu Dhabi: 67% men; 48% nationals; 33%: >60 years old)	Cost data: official list of charges/rates for patients not covered by health insurance Resources use: interviewer-administered questionnaire completed by patients	Components: direct costs (visits to primary health care centers or diabetes clinic, laboratory tests, medications, hospitalizations due to diabetes complications, and emergency room visits due to diabetes) Approach: marco-costing	Health care payer	1 year	No complications: US$1,605 ($2,015 adjusted to 2015) Microvascular complications: US$3,453 ($4,334 adjusted to 2015) Macrovascular complications: US$10,300 ($12,929 adjusted to 2015) Micro- and macrovascular complications: US$15,104 ($18,959 adjusted to 2015) Cost drivers: diabetes-related complications; treatment with insulin	Self-reported questionnaire, uncertainties not thoroughly addressed, and sensitivity analyses not conducted

## Abbreviations

UAE: United Arab Emirates
NCDs: Noncommunicable diseases
PROSPERO: International Prospective Register of Systematic Reviews
CINAHL: Cumulative Index to Nursing and Allied Health Literature
IMEMR: Index Medicus for the Eastern Mediterranean Region
PQDT: ProQuest Dissertations & Theses Database
OATD: Open Access Theses and Dissertations
IDF: International Diabetes Federation
COI: Cost of illness
NCEP: National Cholesterol Education Adult Panel III
HDL-C: High-density lipoprotein cholesterol
IFG: Impaired fasting glucose
ASR: Age-standardized rate
PY: Person-years
BMI: Body mass index
WHO: World Health Organization
US: United States
LDL-C: Low-density lipoprotein cholesterol
DHA: Dubai Health Authority
DSC: Dubai Statistics Center
HAAD: Health Authority of Abu Dhabi.

## Supplementary Materials

Supplementary Materials Appendix A: search strategy followed and flow chart. Appendix B: quality assessment of the cost of illness for diabetes in the United Arab Emirates study.
