# Supplementary material for: The Epidemiology and Economic Burden of Obesity and Related Cardiometabolic Disorders in the United Arab Emirates: A Systematic Review and Qualitative Synthesis
Source: J Obes. 2018 Dec 3;2018:2185942. doi: 10.1155/2018/2185942 (PMC6311818; doi:10.1155/2018/2185942)
Supplement: Supplementary Materials — Appendix A: search strategy followed and flow chart. Appendix B: quality assessment of the cost of illness for diabetes in the United Arab Emirates study. [file 2185942.f1.zip › Appendix A- Search strategy and Flow Chart.docx]

***Appendix A-a:*** Search strategy

**Cumulative Index to Nursing and Allied Health Literature (CINAHL)**

| **#** | **Query** |
| --- | --- |
| S20 | S17 AND S18 AND S19 |
| S19 | S13 OR S14 OR S15 OR S16 |
| S18 | S10 OR S11 OR S12 |
| S17 | S1 OR S2 OR S3 OR S4 OR S5 OR S6 OR S7 OR S8 OR S9 |
| S16 | TI (((quality OR value) N2 life) OR QOL* OR HRQOL* OR HR-QOL*) OR AB (((quality OR value) N2 life) OR QOL* OR HRQOL* OR HR-QOL*) OR TI (((life N1 year*) OR LY* OR QALY*) N2 (lost OR loss*)) OR AB (((life N1 year*) OR LY* OR QALY*) N2 (lost OR loss*)) OR TI DALY* OR AB DALY* |
| S15 | TI (cost* OR price* OR pricing* OR burden OR economic*) OR AB (cost* OR price* OR pricing* OR burden OR economic*) OR TI (human N1 capital) OR AB (human N1 capital) OR TI (Health N2 care N2 (cost* OR spending OR expenditure*)) OR AB (Health N2 care N2 (cost* OR spending OR expenditure*)) OR TI (resource* N3 (utiliz* OR utilis* OR intensive OR consum*)) OR AB (resource* N3 (utiliz* OR utilis* OR intensive OR consum*)) OR TI (Expenditure* OR budget* OR (value N1 (money or monetary)) OR fee OR fees) OR AB (Expenditure* OR budget* OR (value N1 (money or monetary)) OR fee OR fees) |
| S14 | TI (Inciden* OR prevalen* OR occuren* OR epidemiolog* OR mortalit* OR disabilit* OR dis-abilit* OR dysfunction* OR dys-function* OR death* OR morbidit* OR morbimortalit* OR morbi-mortalit* OR survey* OR surveill* OR rate* OR trend*) OR AB (Inciden* OR prevalen* OR occuren* OR epidemiolog* OR mortalit* OR disabilit* OR dis-abilit* OR dysfunction* OR dys-function* OR death* OR morbidit* OR morbimortalit* OR morbi-mortalit* OR survey* OR surveill* OR rate* OR trend*) |
| S13 | (MH "Epidemiology+") OR (MH "Morbidity+") OR (MH "Mortality+") OR (MH "Epidemiological Research") OR (MH "Economics") OR (MH "Costs and Cost Analysis+") OR (MH "Economic Aspects of Illness") OR (MH "Economic Value of Life") OR (MH "Resource Allocation+") OR (MH "Quality-Adjusted Life Years") OR (MH "Quality of Life") |
| S12 | TI (dubai OR abudhabi OR (abu N1 dhabi) OR ajman OR fujaira* OR sharja* OR sharga* OR sharka* OR (ras N1 (alkhaima* OR elkhaima* OR ((al OR el) N1 khaima*))) OR ((um* OR om*) N1 ((al OR el) N1 (qaiwain OR quwain) OR alqaiwain OR alquwain OR elqaiwain OR elquwain)) OR AB (dubai OR abudhabi OR (abu N1 dhabi) OR ajman OR fujaira* OR sharja* OR sharga* OR sharka* OR (ras N1 (alkhaima* OR elkhaima* OR ((al OR el) N1 khaima*))) OR ((um* OR om*) N1 ((al OR el) N1 (qaiwain OR quwain) OR alqaiwain OR alquwain OR elqaiwain OR elquwain)) |
| S11 | TI (emirat* OR UAE OR bedouin* OR gulf OR (trucial N1 state*)) OR AB (emirat* OR UAE OR bedouin* OR gulf OR (trucial N1 state*)) |
| S10 | (MH "United Arab Emirates") OR (MH "Arabs") |
| S9 | TI (cancer* OR tumo#r* OR neoplas* OR metasta* OR oncolog* OR leuk#emi* OR carcinoma* OR adenocarcinom* OR adeno-carcinom* OR lymphoma*) OR AB (cancer* OR tumo#r* OR neoplas* OR metasta* OR oncolog* OR leuk#emi* OR carcinoma* OR adenocarcinom* OR adeno-carcinom* OR lymphoma*) |
| S8 | TI ((high* OR elevat* OR increas* OR borderline* OR border-line*) N3 (glucose OR glyc#emi* OR sugar OR cholesterol* OR lipid* OR triglyceride* OR fat OR fats OR TG OR LDL OR lipo-protein* OR lipoprotein*)) OR AB ((high* OR elevat* OR increas* OR borderline* OR border-line*) N3 (glucose OR glyc#emi* OR sugar OR cholesterol* OR lipid* OR triglyceride* OR fat OR fats OR TG OR LDL OR lipo-protein* OR lipoprotein*)) OR TI ((low* OR decreas* OR suboptimal OR sub-optimal OR reduc*) N3 (lipo-protein* OR lipoprotein* OR HDL)) OR AB ((low* OR decreas* OR suboptimal OR sub-optimal OR reduc*) N3 (lipo-protein* OR lipoprotein* OR HDL)) OR TI (dyslipid#emia* OR hyperlipid#emi* OR hyper-lipid#emi* OR hypertriglycerid#emi* OR hyper-triglycerid#emi* OR hypercholesterol#emi* OR hyper-cholesterol#emi*) OR AB (dyslipid#emia* OR hyperlipid#emi* OR hyper-lipid#emi* OR hypertriglycerid#emi* OR hyper-triglycerid#emi* OR hypercholesterol#emi* OR hyper-cholesterol#emi*) |
| S7 | TI (Stroke* OR CVA) OR AB (Stroke* OR CVA) OR TI (hypertensi* OR HTN OR ((elevat* OR high* OR increas*) N2 blood N1 pressure*) OR SBP OR DBP OR HBP) OR AB (hypertensi* OR HTN OR ((elevat* OR high* OR increas*) N2 blood N1 pressure*) OR SBP OR DBP OR HBP) |
| S6 | TI (diabet* OR pre-diabet* OR prediabet* OR DM OR T2D OR T1D OR NIDD OR IDD OR hyperglyc#emi* OR hyper-glyc#emi* OR dysglyc#emi* OR dys-glyc#emi*) OR AB (diabet* OR pre-diabet* OR prediabet* OR DM OR T2D OR T1D OR NIDD OR IDD OR hyperglyc#emi* OR hyper-glyc#emi* OR dysglyc#emi* OR dys-glyc#emi*) OR TI ((insulin* N2 resistan*) OR hyperinsulin* OR hyper-insulin* OR hypoinsulin* OR hypo-insulin*) OR AB ((insulin* N2 resistan*) OR hyperinsulin* OR hyper-insulin* OR hypoinsulin* OR hypo-insulin*) OR TI ((syndrome N1 x) OR MetS OR (Met N1 S)) OR AB ((syndrome N1 x) OR MetS OR (Met N1 S)) OR TI ((glyc#emi* OR glucose OR sugar) N3 (control* OR manag* OR regulat* OR surveillan* OR monitor* OR test*)) OR AB ((glyc#emi* OR glucose OR sugar) N3 (control* OR manag* OR regulat* OR surveillan* OR monitor* OR test*)) OR TI (HbA1c OR A1c OR (glycosylated N1 h#emoglobin*)) OR AB (HbA1c OR A1c OR (glycosylated N1 h#emoglobin*)) OR TI (HOMA OR ((homeostatic OR homeo-static) N1 model* N1 assessment*)) OR AB (HOMA OR ((homeostatic OR homeo-static) N1 model* N1 assessment*)) OR TI (Ketoacid* OR (keto N1 acid*)) OR AB (Ketoacid* OR (keto N1 acid*)) |
| S5 | TI ((cardiometabolic OR (cardio N1 metabolic) OR metabolic OR dysmetabolic OR dys-metabolic OR nutrition*) N3 (disease* OR disorder* OR syndrome* OR illness* OR condition* OR abnormalit*)) OR AB ((cardiometabolic OR (cardio N1 metabolic) OR metabolic OR dysmetabolic OR dys-metabolic OR nutrition*) N3 (disease* OR disorder* OR syndrome* OR illness* OR condition* OR abnormalit*)) OR TI ((heart OR cardiac OR cardiovascular OR (cardio N1 vascular) OR vascular OR myocardi*) N2 (disease* OR failure* OR attack* OR disorder* OR shock* OR syndrome* OR infarct* OR condition* OR abnormalit*)) OR AB ((heart OR cardiac OR cardiovascular OR (cardio N1 vascular) OR vascular OR myocardi*) N2 (disease* OR failure* OR attack* OR disorder* OR syndrome* OR shock* OR infarct* OR condition* OR abnormalit*)) OR TI (((coronary OR congestive OR (right N1 sided) OR (left N1 sided)) N3 (failure* OR disease* OR illness* OR syndrome OR disorder*)) OR CHF OR CHD OR CVD*) OR AB (((coronary OR congestive OR (right N1 sided) OR (left N1 sided)) N3 (failure* OR disease* OR illness* OR syndrome OR disorder*)) OR CHF OR CHD OR CVD*) |
| S4 | TI ((waist OR abdominal OR abdomen*) N1 (circumference* OR size* OR adiposit*)) OR AB ((waist OR abdominal OR abdomen*) N1 (circumference* OR size* OR adiposit*)) OR TI (BMI OR (body N1 mass N2 (index* OR indices*))) OR AB (BMI OR (body N1 mass N2 (index* OR indices*))) OR TI ((over N1 nutrition*) OR overnutrition* OR (body N2 (size OR weight))) OR AB ((over N1 nutrition*) OR overnutrition* OR (body N2 (size OR weight))) |
| S3 | TI (obes* OR (over N1 weight*) OR overweight* OR (weight N3 (gain* OR increas*))) OR AB (obes* OR (over N1 weight*) OR overweight* OR (weight N3 (gain* OR increas*))) |
| S2 | TI (((chronic OR (long N1 term) OR continu*) N3 (disease* OR illness* OR condition* OR syndrome* OR disorder* OR failure* OR care OR caring) OR NCD* OR ((non N1 communicable) OR noncommunicable) N1 (disease* OR illness*))) OR AB (((chronic OR (long N1 term) OR continu*) N3 (disease* OR illness* OR condition* OR syndrome* OR disorder* OR failure* OR care OR caring) OR NCD* OR ((non N1 communicable) OR noncommunicable) N1 (disease* OR illness*))) |
| S1 | (MH "Chronic Disease") OR (MH "Nutrition Disorders") OR (MH "Metabolic Diseases") OR (MH "Obesity+") OR (MH "Weight Gain+") OR (MH "Hyperphagia") OR (MH "Body Weight") OR (MH "Waist Circumference") OR (MH "Body Size") OR (MH "Body Mass Index") OR (MH "Abdominal Fat") OR (MH "Adipose Tissue Distribution") OR (MH "Cardiovascular Diseases") OR (MH "Heart Diseases+") OR (MH "Vascular Diseases+") OR (MH "Diabetes Mellitus+") OR (MH "Glucose Metabolism Disorders") OR (MH "Hyperglycemia+") OR (MH "Blood Glucose Monitoring+") OR (MH "Blood Glucose") OR (MH "Hyperinsulinism+") OR (MH "Blood Pressure+") OR (MH "Triglycerides") OR (MH "Cholesterol+") OR (MH "Neoplasms+") |

**EMBASE**

1 exp Chronic Disease/ or nutrition disorders/ or exp overnutrition/ or exp overweight/ or body size/ or exp waist circumference/ or exp Body Fat Distribution/ or body mass index/ or cardiovascular diseases/ or exp heart diseases/ or exp vascular diseases/ or metabolic diseases/ or exp Insulin Resistance/ or glucose metabolism disorders/ or diabetes mellitus/ or exp diabetes mellitus, type 1/ or exp diabetes mellitus, type 2/ or Diabetic Ketoacidosis/ or exp prediabetic state/ or Blood Glucose/ or exp hyperglycemia/ or Insulin/ or hyperinsulinism/ or exp Stroke/ or exp Hypertension/ or Blood Pressure/ or dyslipidemias/ or exp hyperlipidemias/ or exp lipoproteins, hdl/ or exp lipoproteins, ldl/ or Triglycerides/ or Cholesterol/ or exp Neoplasms/

2 (((chronic or (long adj term) or continu*) adj3 (disease* or illness* or condition* or syndrome* or disorder* or failure* or care or caring)) or NCD* or (((non adj communicable) or noncommunicable) adj (disease* or illness*))).ti,ab,sh.

3 (obes* or (over adj weight*) or overweight* or (weight adj3 (gain* or increas*))).ti,ab,sh.

4 ((waist or abdominal or abdomen*) adj (circumference* or size* or adiposit*)).ti,ab,sh.

5 (BMI or (body adj mass adj2 (index* or indices))).ti,ab,sh.

6 ((over adj nutrition*) or overnutrition* or (body adj2 (size or weight))).ti,ab,sh.

7 ((cardiometabolic or (cardio adj metabolic) or metabolic or dysmetabolic or dys-metabolic or nutrition*) adj3 (disease* or disorder* or syndrome* or illness* or condition* or abnormalit*)).ti,ab,sh.

8 ((heart or cardiac or cardiovascular or (cardio adj vascular) or vascular or myocardi*) adj2 (disease* or failure* or attack* or disorder* or syndrome* or shock* or infarct* or condition* or abnormalit*)).ti,ab,sh.

9 (((coronary or congestive or (right adj sided) or (left adj sided)) adj3 (failure* or disease* or illness* or syndrome or disorder*)) or CHF or CHD or CVD*).ti,ab,sh.

10 (diabet* or pre-diabet* or prediabet* or DM or T2D or T1D or NIDD or IDD or hyperglyc?emi* or hyper-glyc?emi* or dysglyc?emi* or dys-glyc?emi*).ti,ab,sh.

11 ((insulin* adj2 resistan*) or hyperinsulin* or hyper-insulin* or hypoinsulin* or hypo-insulin*).ti,ab,sh.

12 ((syndrome adj x) or MetS or (Met adj S)).ti,ab,sh.

13 ((glyc?emi* or glucose or sugar) adj3 (control* or manag* or regulat* or surveillan* or monitor* or test*)).ti,ab,sh.

14 (HbA1c or A1c or (glycosylated adj h?emoglobin*)).ti,ab,sh.

15 (HOMA or ((homeostatic or homeo-static) adj model* adj assessment*)).ti,ab,sh.

16 (Ketoacid* or (keto adj acid*)).ti,ab,sh.

17 (Stroke* or CVA).ti,ab,sh.

18 (hypertensi* or HTN or ((elevat* or high* or increas*) adj2 blood adj pressure*) or SBP or DBP or HBP).ti,ab,sh.

19 ((high* or elevat* or increas* or borderline* or border-line*) adj3 (glucose or glyc?emi* or sugar or cholesterol* or lipid* or triglyceride* or fat or fats or TG or LDL or lipo-protein* or lipoprotein*)).ti,ab,sh.

20 ((low* or decreas* or suboptimal or sub-optimal or reduc*) adj3 (lipo-protein* or lipoprotein* or HDL)).ti,ab,sh.

21 (dyslipid?emia* or hyperlipid?emi* or hyper-lipid?emi* or hypertriglycerid?emi* or hyper-triglycerid?emi* or hypercholesterol?emi* or hyper-cholesterol?emi*).ti,ab,sh.

22 (cancer* or tumo?r* or neoplas* or metasta* or oncolog* or leuk?emi* or carcinoma* or adenocarcinom* or adeno-carcinom* or lymphoma*).ti,ab,sh.

23 1 or 2 or 3 or 4 or 5 or 6 or 7 or 8 or 9 or 10 or 11 or 12 or 13 or 14 or 15 or 16 or 17 or 18 or 19 or 20 or 21 or 22

24 exp United Arab Emirates/ or exp Arabs/

25 (emirat* or UAE or bedouin* or gulf or (trucial adj state*)).ti,ab,sh.

26 (dubai or abudhabi or (abu adj dhabi) or ajman or fujaira* or sharja* or sharga* or sharka* or (ras adj (alkhaima* or elkhaima* or ((al or el) adj khaima*))) or ((um* or om*) adj (((al or el) adj (qaiwain or quwain)) or alqaiwain or alquwain or elqaiwain or elquwain))).ti,ab,sh.

27 24 or 25 or 26

28 exp incidence/ or exp prevalence/ or exp epidemiological monitoring/ or exp sentinel surveillance/ or exp epidemiologic studies/ or Epidemiology/ or exp morbidity/ or exp mortality/ or health surveys/ or exp population surveillance/ or economics/ or exp "costs and cost analysis"/ or exp economics, medical/ or Quality-adjusted life years/ or quality of life/ or value of life/ or models, economic/

29 (inciden* or prevalen* or occuren* or epidemiolog* or mortalit* or disabilit* or dis-abilit* or dysfunction* or dys-function* or death* or morbidit* or morbimortalit* or morbi-mortalit* or survey* or surveill* or rate* or trend*).ti,ab,sh.

30 (cost* or price* or pricing* or burden or economic*).ti,ab,sh.

31 (human adj capital).ti,ab,sh.

32 (resource* adj3 (utiliz* or utilis* or intensive or consum*)).ti,ab,sh.

33 (expenditure* or budget* or (value adj1 (money or monetary)) or fee or fees).ti,ab,sh.

34 (((quality or value) adj2 life) or QOL* or HRQOL* or HR-QOL*).ti,ab,sh.

35 (((life adj year*) or LY* or QALY*) adj2 (lost or loss*)).ti,ab,sh.

36 DALY*.ti,ab,sh.

37 28 or 29 or 30 or 31 or 32 or 33 or 34 or 35 or 36

38 23 and 27 and 37

**Medline**

1 exp Chronic Disease/ or nutrition disorders/ or exp overnutrition/ or exp overweight/ or body size/ or exp waist circumference/ or exp Body Fat Distribution/ or body mass index/ or cardiovascular diseases/ or exp heart diseases/ or exp vascular diseases/ or metabolic diseases/ or exp Insulin Resistance/ or glucose metabolism disorders/ or diabetes mellitus/ or exp diabetes mellitus, type 1/ or exp diabetes mellitus, type 2/ or Diabetic Ketoacidosis/ or exp prediabetic state/ or Blood Glucose/ or exp hyperglycemia/ or Insulin/ or hyperinsulinism/ or exp Stroke/ or exp Hypertension/ or Blood Pressure/ or dyslipidemias/ or exp hyperlipidemias/ or exp lipoproteins, hdl/ or exp lipoproteins, ldl/ or Triglycerides/ or Cholesterol/ or exp Neoplasms/

2 (((chronic or (long adj term) or continu*) adj3 (disease* or illness* or condition* or syndrome* or disorder* or failure* or care or caring)) or NCD* or (((non adj communicable) or noncommunicable) adj (disease* or illness*))).ti,ab,sh.

3 (obes* or (over adj weight*) or overweight* or (weight adj3 (gain* or increas*))).ti,ab,sh.

4 ((waist or abdominal or abdomen*) adj (circumference* or size* or adiposit*)).ti,ab,sh.

5 (BMI or (body adj mass adj2 (index* or indices))).ti,ab,sh.

6 ((over adj nutrition*) or overnutrition* or (body adj2 (size or weight))).ti,ab,sh.

7 ((cardiometabolic or (cardio adj metabolic) or metabolic or dysmetabolic or dys-metabolic or nutrition*) adj3 (disease* or disorder* or syndrome* or illness* or condition* or abnormalit*)).ti,ab,sh.

8 ((heart or cardiac or cardiovascular or (cardio adj vascular) or vascular or myocardi*) adj2 (disease* or failure* or attack* or disorder* or syndrome* or shock* or infarct* or condition* or abnormalit*)).ti,ab,sh.

9 (((coronary or congestive or (right adj sided) or (left adj sided)) adj3 (failure* or disease* or illness* or syndrome or disorder*)) or CHF or CHD or CVD*).ti,ab,sh.

10 (diabet* or pre-diabet* or prediabet* or DM or T2D or T1D or NIDD or IDD or hyperglyc?emi* or hyper-glyc?emi* or dysglyc?emi* or dys-glyc?emi*).ti,ab,sh.

11 ((insulin* adj2 resistan*) or hyperinsulin* or hyper-insulin* or hypoinsulin* or hypo-insulin*).ti,ab,sh.

12 ((syndrome adj x) or MetS or (Met adj S)).ti,ab,sh.

13 ((glyc?emi* or glucose or sugar) adj3 (control* or manag* or regulat* or surveillan* or monitor* or test*)).ti,ab,sh.

14 (HbA1c or A1c or (glycosylated adj h?emoglobin*)).ti,ab,sh.

15 (HOMA or ((homeostatic or homeo-static) adj model* adj assessment*)).ti,ab,sh.

16 (Ketoacid* or (keto adj acid*)).ti,ab,sh.

17 (Stroke* or CVA).ti,ab,sh.

18 (hypertensi* or HTN or ((elevat* or high* or increas*) adj2 blood adj pressure*) or SBP or DBP or HBP).ti,ab,sh.

19 ((high* or elevat* or increas* or borderline* or border-line*) adj3 (glucose or glyc?emi* or sugar or cholesterol* or lipid* or triglyceride* or fat or fats or TG or LDL or lipo-protein* or lipoprotein*)).ti,ab,sh.

20 ((low* or decreas* or suboptimal or sub-optimal or reduc*) adj3 (lipo-protein* or lipoprotein* or HDL)).ti,ab,sh.

21 (dyslipid?emia* or hyperlipid?emi* or hyper-lipid?emi* or hypertriglycerid?emi* or hyper-triglycerid?emi* or hypercholesterol?emi* or hyper-cholesterol?emi*).ti,ab,sh.

22 (cancer* or tumo?r* or neoplas* or metasta* or oncolog* or leuk?emi* or carcinoma* or adenocarcinom* or adeno-carcinom* or lymphoma*).ti,ab,sh.

23 1 or 2 or 3 or 4 or 5 or 6 or 7 or 8 or 9 or 10 or 11 or 12 or 13 or 14 or 15 or 16 or 17 or 18 or 19 or 20 or 21 or 22

24 exp United Arab Emirates/ or exp Arabs/

25 (emirat* or UAE or bedouin* or gulf or (trucial adj state*)).ti,ab,sh.

26 (dubai or abudhabi or (abu adj dhabi) or ajman or fujaira* or sharja* or sharga* or sharka* or (ras adj (alkhaima* or elkhaima* or ((al or el) adj khaima*))) or ((um* or om*) adj (((al or el) adj (qaiwain or quwain)) or alqaiwain or alquwain or elqaiwain or elquwain))).ti,ab,sh.

27 24 or 25 or 26

28 exp incidence/ or exp prevalence/ or exp epidemiological monitoring/ or exp sentinel surveillance/ or exp epidemiologic studies/ or Epidemiology/ or exp morbidity/ or exp mortality/ or health surveys/ or exp population surveillance/ or economics/ or exp "costs and cost analysis"/ or exp economics, medical/ or Quality-adjusted life years/ or quality of life/ or value of life/ or models, economic/

29 (inciden* or prevalen* or occuren* or epidemiolog* or mortalit* or disabilit* or dis-abilit* or dysfunction* or dys-function* or death* or morbidit* or morbimortalit* or morbi-mortalit* or survey* or surveill* or rate* or trend*).ti,ab,sh.

30 (cost* or price* or pricing* or burden or economic*).ti,ab,sh.

31 (human adj capital).ti,ab,sh.

32 (resource* adj3 (utiliz* or utilis* or intensive or consum*)).ti,ab,sh.

33 (expenditure* or budget* or (value adj1 (money or monetary)) or fee or fees).ti,ab,sh.

34 (((quality or value) adj2 life) or QOL* or HRQOL* or HR-QOL*).ti,ab,sh.

35 (((life adj year*) or LY* or QALY*) adj2 (lost or loss*)).ti,ab,sh.

36 DALY*.ti,ab,sh.

37 epidemiology.fs.

38 economics.fs.

39 trends.fs.

40 28 or 29 or 30 or 31 or 32 or 33 or 34 or 35 or 36 or 37 or 38 or 39

41 23 and 27 and 40

**PubMed**

| #20 | Search (#17 AND #18 AND #19) |
| --- | --- |
| #19 | Search (#13 OR #14 OR #15 OR #16) |
| #18 | Search (#10 OR #11 OR #12) |
| #17 | Search (#1 OR #2 OR #3 OR #4 OR #5 OR #6 OR #7 OR #8 OR #9) |
| #16 | Search (Quality of life[tw] OR quality-of-life[tw] OR value of life[tw] OR value-of-life[tw] OR QOL[tw] OR HRQOL[tw] OR HR-QOL[tw] OR life year lost[tw] OR life-year lost[tw] OR life years lost[tw] OR life-years lost[tw] OR LY lost[tw] OR QALY lost[tw] OR life year loss[tw] OR life-year loss[tw] OR LY loss[tw] OR QALY loss[tw] OR DALY[tw]) |
| #15 | Search (Cost[tw] OR costs[tw] OR costing[tw] OR price[tw] OR prices[tw] OR pricing*[tw] OR burden[tw] OR economic*[tw] OR human capital*[tw] OR resource utilization[tw] OR resource utilisation[tw] OR resources utilization[tw] OR resources utilisation[tw] OR resource intensive[tw] OR resource consuming[tw] OR resource-intensive[tw] OR resource-consuming[tw] OR expenditure*[tw] OR budget*[tw] OR value money[tw] OR value monetary[tw] OR fee[tw] OR fees[tw]) |
| #14 | Search (Inciden*[tw] OR prevalen*[tw] OR occuren*[tw] OR epidemiolog*[tw] OR mortalit*[tw] OR disabilit*[tw] OR dis-abilit*[tw] OR dysfunction*[tw] OR dys-function*[tw] OR death*[tw] OR morbidit*[tw] OR morbimortalit*[tw] OR morbi-mortalit*[tw] OR survey*[tw] OR surveill*[tw] OR rate[tw] OR rates[tw] OR trend[tw] OR trends[tw]) |
| #13 | Search (incidence[mesh] OR prevalence[mesh] OR epidemiological monitoring[mesh] OR sentinel surveillance[mesh] OR epidemiologic studies[mesh] OR Epidemiology[mesh: noexp] OR morbiditY[mesh] OR mortality[mesh] OR health surveys[mesh: noexp] OR population surveillance[mesh] OR economics[mesh: noexp] OR "costs and cost analysis"[mesh] OR economics, medical[mesh] OR Quality-adjusted life years[mesh: noexp] OR quality of life[mesh: noexp] OR value of life[mesh: noexp] OR models, economic[mesh: noexp]) |
| #12 | Search (Dubai[tw]OR abudhabi[tw] OR abu dhabi[tw] OR ajman[tw] OR fujaira*[tw] OR sharja*[tw] OR sharga*[tw] OR sharka*[tw] OR ras alkhaima*[tw] OR ras elkhaima*[tw] OR ras al khaima*[tw] OR ras el khaima*[tw] OR umm al qaiwain[tw] OR umm el qaiwain[tw] OR umm al quwain[tw] OR umm el quwain[tw] OR umm alqaiwain[tw] OR umm elqaiwain[tw] OR om alqaiwain[tw] OR om elqaiwain[tw] OR umm alqaiwain[tw] OR umm elqaiwain[tw] OR om alqaiwain[tw] OR om elqaiwain[tw] OR umm alquwain[tw] OR umm elquwain[tw] OR om alquwain[tw] OR om elquwain[tw]) |
| #11 | Search (Emirat*[tw] OR UAE[tw] OR bedouin*[tw] OR gulf[tw] OR trucial state*[tw]) |
| #10 | Search (United Arab Emirates[mesh] OR Arabs[mesh]) |
| #9 | Search (Cancer[tw] OR cancers[tw] OR cancerous[tw] OR tumor[tw] OR tumors[tw] OR tumour[tw] OR tumours[tw] OR neoplas*[tw] OR metasta*[tw] OR oncolog*[tw] OR leukemi*[tw] OR leukaemia*[tw] OR carcinoma*[tw] OR adenocarcinom*[tw] OR adeno-carcinom*[tw] OR lymphoma*[tw]) |
| #8 | Search (High cholesterol*[tw] OR elevated cholesterol*[tw] OR increased cholesterol*[tw] OR borderline cholesterol*[tw] OR border-line cholesterol*[tw] OR high lipid*[tw] OR elevated lipid*[tw] OR increased lipid*[tw] OR borderline lipid*[tw] OR border-line lipid*[tw] OR high triglyceride*[tw] OR elevated triglyceride*[tw] OR increased triglyceride*[tw] OR borderline triglyceride*[tw] OR border-line triglyceride*[tw] OR high fat[tw] OR elevated fat[tw] OR increased fat[tw] OR borderline fat[tw] OR border-line fat[tw] OR high TG[tw] OR elevated TG[tw] OR increased TG[tw] OR borderline TG[tw] OR border-line TG[tw] OR high LDL[tw] OR elevated LDL[tw] OR increased LDL[tw] OR borderline LDL[tw] OR border-line LDL[tw] OR dyslipidemia*[tw] OR dyslipidaemia*[tw] OR hyperlipidemi*[tw] OR hyperlipidaemi*[tw] OR hyper-lipidemi*[tw] OR hyper-lipidaemi*[tw] OR hypertriglyceridemi*[tw] OR hypertriglyceridaemi*[tw] OR hyper-triglyceridemi*[tw] OR hyper-triglyceridaemi*[tw] OR hypercholesterolemi*[tw] OR hypercholesterolaemi*[tw] OR hyper-cholesterolemi*[tw] OR hyper-cholesterolaemi*[tw] OR lipo-protein*[tw] OR lipoprotein*[tw] OR low HDL[tw] OR decreased HDL[tw] OR suboptimal HDL[tw] OR sub-optimal HDL[tw] OR reduced HDL[tw]) |
| #7 | Search (Stroke*[tw] OR CVA[tw] OR Hypertension[tw] OR hypertensive[tw] OR HTN[tw] OR elevated blood pressure[tw] OR high blood pressure[tw] OR increased blood pressure[tw] OR SBP[tw] OR DBP[tw] OR HBP[tw]) |
| #6 | Search (Diabetic*[tw] OR diabetes[tw] OR pre-diabetes[tw] OR pre-diabetic*[tw] OR prediabetes[tw] OR prediabetic*[tw] OR DM[tw] OR T2D[tw] OR T1D[tw] OR NIDD[tw] OR IDD[tw] OR hyperglycemic*[tw] OR hyperglycemia*[tw] OR hyperglycaemic*[tw] OR hyperglycaemia*[tw] OR hyper-glycemic*[tw] OR hyper-glycemia*[tw] OR hyperglycaemic*[tw] OR hyperglycaemia*[tw] OR dysglycemic*[tw] OR dysglycemia*[tw] OR dysglycaemic*[tw] OR dysglycaemia*[tw] OR dys-glycemic*[tw] OR dys-glycemia*[tw] OR dys-glycaemic*[tw] OR dys-glycaemia*[tw] OR Insulin resistan*[tw] OR hyperinsulin*[tw] OR hyper-insulin*[tw] OR hypoinsulin*[tw] OR hypo-insulin*[tw] OR Syndrome X[tw] OR MetS[tw] OR Met S[tw] OR Glycemia*[tw] OR glycaemia*[tw] OR glycemic*[tw] OR glycaemic*[tw] OR glucose[tw] OR sugar[tw] OR HbA1c[tw] OR A1c[tw] OR glycosylated hemoglobin*[tw] OR glycosylated haemoglobin*[tw] OR Ketoacid*[tw] OR keto-acid*[tw] OR HOMA[tw] OR homeostatic model assessment[tw] OR homeo-static model assessment[tw]) |
| #5 | Search (Heart disease*[tw] OR heart failure*[tw] OR heart attack*[tw] OR heart disorder*[tw] OR heart condition*[tw] OR heart abnormalit*[tw] OR cardiac disease*[tw] OR cardiac failure*[tw] OR cardiac attack*[tw] OR cardiac disorder*[tw] OR cardiac condition*[tw] OR cardiac abnormalit*[tw] OR cardiovascular disease*[tw] OR cardiovascular disorder*[tw] OR cardiovascular condition*[tw] OR cardiovascular abnormalit*[tw] OR cardio-vascular disease*[tw] OR cardio-vascular disorder*[tw] OR cardio-vascular condition*[tw] OR cardio-vascular abnormalit*[tw] OR vascular disease*[tw] OR vascular disorder*[tw] OR vascular condition*[tw] OR vascular abnormalit*[tw] OR myocardium failure*[tw] OR myocardium disorder*[tw] OR myocardium abnormalit*[tw] OR myocardial infarct*[tw] OR myocardial failure*[tw] OR CHF[tw] OR CHD[tw] OR CVD*[tw] OR Coronary failure*[tw] OR coronary disease*[tw] OR coronary illness*[tw] OR coronary syndrome*[tw] OR coronary disorder*[tw] OR congestive illness*[tw]) |
| #4 | Search (Cardiometabolic disease*[tw] OR cardiometabolic disorder*[tw] OR cardiometabolic syndrome*[tw] OR cardiometabolic illness*[tw] OR cardiometabolic condition*[tw] OR cardiometabolic abnormalit*[tw] OR cardio-metabolic disease*[tw] OR cardio-metabolic disorder*[tw] OR cardio-metabolic syndrome*[tw] OR cardio-metabolic illness*[tw] OR cardio-metabolic condition*[tw] OR cardio-metabolic abnormalit*[tw] OR metabolic disease*[tw] OR metabolic disorder*[tw] OR metabolic syndrome*[tw] OR metabolic illness*[tw] OR metabolic condition*[tw] OR metabolic abnormalit*[tw] OR dysmetabolic disease*[tw] OR dysmetabolic disorder*[tw] OR dysmetabolic syndrome*[tw] OR dysmetabolic illness*[tw] OR dysmetabolic condition*[tw] OR dysmetabolic abnormalit*[tw] OR dys-metabolic disease*[tw] OR dys-metabolic disorder*[tw] OR dys-metabolic syndrome*[tw] OR dys-metabolic illness*[tw] OR dys-metabolic condition*[tw] OR dys-metabolic abnormalit*[tw] OR nutrition disease*[tw] OR nutrition disorder*[tw] OR nutrition syndrome*[tw] OR nutrition illness*[tw] OR nutrition condition*[tw] OR nutrition abnormalit*[tw] OR nutritional disease*[tw] OR nutritional disorder*[tw] OR nutritional syndrome*[tw] OR nutritional illness*[tw] OR nutritional condition*[tw] OR nutritional abnormalit*[tw]) |
| #3 | Search (Obese[tw] OR obesity[tw] OR over-weight*[tw] OR overweight*[tw] OR weight gain*[tw] OR weight increas*[tw] OR waist circumference*[tw] OR waist size*[tw] OR waist adiposit*[tw] OR abdominal circumference*[tw] OR abdominal size*[tw] OR abdominal adiposit*[tw] OR abdomen circumference*[tw] OR abdomen size*[tw] OR abdomen adiposit*[tw] OR BMI[tw] OR body mass index*[tw] OR body mass indices[tw] OR over-nutrition*[tw] OR overnutrition*[tw] OR body size*[tw] OR body weight*[tw]) |
| #2 | Search (Chronic disease*[tw] OR chronic illness*[tw] OR chronic condition*[tw] OR chronic failure[tw] OR chronic care[tw] Long term disease*[tw] OR long term illness*[tw] OR long term condition*[tw] OR long term care[tw] OR continuous disease*[tw] OR continuous illness*[tw] OR continuous condition*[tw] OR continuous care[tw] OR continuing disease*[tw] OR continuing illness*[tw] OR continuing condition*[tw] OR continuing care[tw] OR NCD*[tw] OR non communicable disease*[tw] OR non communicable illness*[tw] OR noncommunicable disease*[tw] OR noncommunicable illness*[tw]) |
| #1 | Search (Chronic Disease[mesh] OR nutrition disorders[mesh: noexp] OR overnutrition[mesh] OR overweight[mesh] OR body size[mesh: noexp] OR waist circumference[mesh] OR Body Fat Distribution[mesh] OR body mass index[mesh: noexp] OR cardiovascular diseases[mesh: noexp] OR heart diseases[mesh] OR vascular diseases[mesh] OR metabolic diseases[mesh: noexp] OR Insulin Resistance[mesh] OR glucose metabolism disorders[mesh: noexp] OR diabetes mellitus[mesh: noexp] OR diabetes mellitus, type 1[mesh] OR diabetes mellitus, type 2[mesh] OR Diabetic Ketoacidosis[mesh: noexp] OR prediabetic state[mesh] OR Blood Glucose[mesh: noexp] OR hyperglycemia[mesh] OR Insulin[mesh: noexp] OR hyperinsulinism[mesh: noexp] OR Stroke[mesh] OR Hypertension[mesh] OR Blood Pressure[mesh: noexp] OR dyslipidemias[mesh: noexp] OR hyperlipidemias[mesh] OR lipoproteins, hdl[mesh] OR lipoproteins, ldl[mesh] OR Triglycerides[mesh: noexp] OR Cholesterol[mesh: noexp] OR Neoplasms[mesh]) |

**Web of Science**

| # 16 | #15 AND #14 AND #13  Indexes=SCI-EXPANDED, SSCI, A&HCI Timespan=All years |
| --- | --- |
| # 15 | #12 OR #11 OR #10  Indexes=SCI-EXPANDED, SSCI, A&HCI Timespan=All years |
| # 14 | #9 OR #8  Indexes=SCI-EXPANDED, SSCI, A&HCI Timespan=All years |
| # 13 | #7 OR #6 OR #5 OR #4 OR #3 OR #2 OR #1  Indexes=SCI-EXPANDED, SSCI, A&HCI Timespan=All years |
| # 12 | TI=(((quality OR value) NEAR/2 life) OR QOL* OR HRQOL* OR HR-QOL*) OR TS=(((quality OR value) NEAR/2 life) OR QOL* OR HRQOL* OR HR-QOL*) OR TI=(((life NEAR/1 year*) OR QALY*) NEAR/2 (lost OR loss*)) OR TS=(((life NEAR/1 year*) OR QALY*) NEAR/2 (lost OR loss*)) OR TI=DALY* OR TS=DALY*  Indexes=SCI-EXPANDED, SSCI, A&HCI Timespan=All years |
| # 11 | TI=(cost* OR price* OR pricing* OR burden OR economic*) OR TS=(cost* OR price* OR pricing* OR burden OR economic*) OR TI=(human NEAR/1 capital) OR TS=(human NEAR/1 capital) OR TI=(Health NEAR/2 care NEAR/2 (cost* OR spending OR expenditure*)) OR TS=(Health NEAR/2 care NEAR/2 (cost* OR spending OR expenditure*)) OR TI=(resource* NEAR/3 (utiliz* OR utilis* OR intensive OR consum*)) OR TS=(resource* NEAR/3 (utiliz* OR utilis* OR intensive OR consum*)) OR TI=(Expenditure* OR budget* OR (value NEAR/1 (money or monetary)) OR fee OR fees) OR TS=(Expenditure* OR budget* OR (value NEAR/1 (money or monetary)) OR fee OR fees)  Indexes=SCI-EXPANDED, SSCI, A&HCI Timespan=All years |
| # 10 | TI=(Inciden* OR prevalen* OR occuren* OR epidemiolog* OR mortalit* OR disabilit* OR dis-abilit* OR dysfunction* OR dys-function* OR death* OR morbidit* OR morbimortalit* OR morbi-mortalit* OR survey* OR surveill* OR rate* OR trend*) OR TS=(Inciden* OR prevalen* OR occuren* OR epidemiolog* OR mortalit* OR disabilit* OR dis-abilit* OR dysfunction* OR dys-function* OR death* OR morbidit* OR morbimortalit* OR morbi-mortalit* OR survey* OR surveill* OR rate* OR trend*)  Indexes=SCI-EXPANDED, SSCI, A&HCI Timespan=All years |
| # 9 | TI=(dubai OR abudhabi OR (abu NEAR/1 dhabi) OR ajman OR fujaira* OR sharja* OR sharga* OR sharka* OR ras-al-khaima* OR ras-el-khaima* OR umm-al-qaiwain OR umm-el-quwain OR umm-al-qaiwain OR umm-al-quwain OR umm-el-qaiwain OR umm-el-quwain OR omm-al-qaiwain OR omm-el-quwain OR omm-al-qaiwain OR omm-al-quwain OR omm-el-qaiwain OR omm-el-quwain) OR TS=(dubai OR abudhabi OR (abu NEAR/1 dhabi) OR ajman OR fujaira* OR sharja* OR sharga* OR sharka* OR ras-al-khaima* OR ras-el-khaima* OR umm-al-qaiwain OR umm-el-quwain OR umm-al-qaiwain OR umm-al-quwain OR umm-el-qaiwain OR umm-el-quwain OR omm-al-qaiwain OR omm-el-quwain OR omm-al-qaiwain OR omm-al-quwain OR omm-el-qaiwain OR omm-el-quwain)  Indexes=SCI-EXPANDED, SSCI, A&HCI Timespan=All years |
| # 8 | TI=(emirat* OR UAE OR bedouin* OR gulf OR (trucial NEAR/1 state*)) OR TS=(emirat* OR UAE OR bedouin* OR gulf OR (trucial NEAR/1 state*))  Indexes=SCI-EXPANDED, SSCI, A&HCI Timespan=All years |
| # 7 | TI=(cancer* OR tumo$r* OR neoplas* OR metasta* OR oncolog* OR leuk$emi* OR carcinoma* OR adenocarcinom* OR adeno-carcinom* OR lymphoma*) OR TS=(cancer* OR tumo$r* OR neoplas* OR metasta* OR oncolog* OR leuk$emi* OR carcinoma* OR adenocarcinom* OR adeno-carcinom* OR lymphoma*)  Indexes=SCI-EXPANDED, SSCI, A&HCI Timespan=All years |
| # 6 | TI=((high* OR elevat* OR increas* OR borderline* OR border-line*) NEAR/3 (glucose OR glyc$emi* OR sugar OR cholesterol* OR lipid* OR triglyceride* OR fat OR fats OR TG OR LDL OR lipo-protein* OR lipoprotein*)) OR TS=((high* OR elevat* OR increas* OR borderline* OR border-line*) NEAR/3 (glucose OR glyc$emi* OR sugar OR cholesterol* OR lipid* OR triglyceride* OR fat OR fats OR TG OR LDL OR lipo-protein* OR lipoprotein*)) OR TI=((low* OR decreas* OR suboptimal OR sub-optimal OR reduc*) NEAR/3 (lipo-protein* OR lipoprotein* OR HDL)) OR TS=((low* OR decreas* OR suboptimal OR sub-optimal OR reduc*) NEAR/3 (lipo-protein* OR lipoprotein* OR HDL)) OR TI=(dyslipid$emia* OR hyperlipid$emi* OR hyper-lipid$emi* OR hypertriglycerid$emi* OR hyper-triglycerid$emi* OR hypercholesterol$emi* OR hyper-cholesterol$emi*) OR TS=(dyslipid$emia* OR hyperlipid$emi* OR hyper-lipid$emi* OR hypertriglycerid$emi* OR hyper-triglycerid$emi* OR hypercholesterol$emi* OR hyper-cholesterol$emi*)  Indexes=SCI-EXPANDED, SSCI, A&HCI Timespan=All years |
| # 5 | TI=(Stroke* OR CVA) OR TS=(Stroke* OR CVA) OR TI=(hypertensi* OR HTN OR ((elevat* OR high* OR increas*) NEAR/2 blood NEAR/1 pressure*) OR SBP OR DBP OR HBP) OR TS=(hypertensi* OR HTN OR ((elevat* OR high* OR increas*) NEAR/2 blood NEAR/1 pressure*) OR SBP OR DBP OR HBP)  Indexes=SCI-EXPANDED, SSCI, A&HCI Timespan=All years |
| # 4 | TI=(diabet* OR pre-diabet* OR prediabet* OR DM OR T2D OR T1D OR NIDD OR IDD OR hyperglyc$emi* OR hyper-glyc$emi* OR dysglyc$emi* OR dys-glyc$emi*) OR TS=(diabet* OR pre-diabet* OR prediabet* OR DM OR T2D OR T1D OR NIDD OR IDD OR hyperglyc$emi* OR hyper-glyc$emi* OR dysglyc$emi* OR dys-glyc$emi*) OR TI=((insulin* NEAR/2 resistan*) OR hyperinsulin* OR hyper-insulin* OR hypoinsulin* OR hypo-insulin*) OR TS=((insulin* NEAR/2 resistan*) OR hyperinsulin* OR hyper-insulin* OR hypoinsulin* OR hypo-insulin*) OR TI=((syndrome NEAR/1 x) OR MetS OR (Met NEAR/1 S)) OR TS=((syndrome NEAR/1 x) OR MetS OR (Met NEAR/1 S)) OR TI=((glyc$emi* OR glucose OR sugar) NEAR/3 (control* OR manag* OR regulat* OR surveillan* OR monitor* OR test*)) OR TS=((glyc$emi* OR glucose OR sugar) NEAR/3 (control* OR manag* OR regulat* OR surveillan* OR monitor* OR test*)) OR TI=(HbA1c OR A1c OR (glycosylated NEAR/1 h$emoglobin*)) OR TS=(HbA1c OR A1c OR (glycosylated NEAR/1 h$emoglobin*)) OR TI=(HOMA OR ((homeostatic OR homeo-static) NEAR/1 model* NEAR/1 assessment*)) OR TS=(HOMA OR ((homeostatic OR homeo-static) NEAR/1 model* NEAR/1 assessment*)) OR TI=(Ketoacid* OR (keto NEAR/1 acid*)) OR TS=(Ketoacid* OR (keto NEAR/1 acid*))  Indexes=SCI-EXPANDED, SSCI, A&HCI Timespan=All years |
| # 3 | TI=((cardiometabolic OR cardio-metabolic OR metabolic OR dysmetabolic OR dys-metabolic OR nutrition*) NEAR/3 (disease* OR disorder* OR syndrome* OR illness* OR condition* OR abnormalit*)) OR TS=((cardiometabolic OR cardio-metabolic OR metabolic OR dysmetabolic OR dys-metabolic OR nutrition*) NEAR/3 (disease* OR disorder* OR syndrome* OR illness* OR condition* OR abnormalit*)) OR TI=((heart OR cardiac OR cardiovascular OR cardio-vascular OR vascular OR myocardi*) N2 (disease* OR failure* OR attack* OR disorder* OR shock* OR syndrome* OR infarct* OR condition* OR abnormalit*)) OR TS=((heart OR cardiac OR cardiovascular OR cardio-vascular OR vascular OR myocardi*) NEAR/2 (disease* OR failure* OR attack* OR disorder* OR syndrome* OR shock* OR infarct* OR condition* OR abnormalit*)) OR TI=(((coronary OR congestive OR right-sided OR left-sided) NEAR/3 (failure* OR disease* OR illness* OR syndrome OR disorder*)) OR CHF OR CHD OR CVD*) OR TS=(((coronary OR congestive OR right-sided OR left-sided) NEAR/3 (failure* OR disease* OR illness* OR syndrome OR disorder*)) OR CHF OR CHD OR CVD*)  Indexes=SCI-EXPANDED, SSCI, A&HCI Timespan=All years |
| # 2 | TI=(obes* OR over-weight* OR overweight* OR (weight NEAR/3 (gain* OR increas*))) OR TS=(obes* OR over-weight* OR overweight* OR (weight NEAR/3 (gain* OR increas*))) OR TI=((waist OR abdominal OR abdomen*) NEAR/1 (circumference* OR size* OR adiposit*)) OR TS=((waist OR abdominal OR abdomen*) NEAR/1 (circumference* OR size* OR adiposit*)) OR TI=(BMI OR (body NEAR/1 mass NEAR/2 (index* OR indices*))) OR TS=(BMI OR (body NEAR/1 mass NEAR/2 (index* OR indices*))) OR TI=(over-nutrition* OR overnutrition* OR (body NEAR/2 (size OR weight))) OR TS=(over-nutrition* OR overnutrition* OR (body NEAR/2 (size OR weight)))  Indexes=SCI-EXPANDED, SSCI, A&HCI Timespan=All years |
| # 1 | TI=(((chronic OR long-term OR continu*) NEAR/3 (disease* OR illness* OR condition* OR syndrome* OR disorder* OR failure* OR care OR caring) OR NCD* OR (non-communicable OR noncommunicable) NEAR/1 (disease* OR illness*))) OR TS=(((chronic OR long-term OR continu*) NEAR/3 (disease* OR illness* OR condition* OR syndrome* OR disorder* OR failure* OR care OR caring) OR NCD* OR (non-communicable OR noncommunicable) NEAR/1 (disease* OR illness*)))  Indexes=SCI-EXPANDED, SSCI, A&HCI Timespan=All years |

**ProQuest Dissertations & Theses Database Open (PQDTO)**

emirates OR emirate OR emirati OR UAE

**International Diabetes Federation**

emirates emirati emirate UAE

**World Bank**

emirates emirati emirate UAE

**Open Access Theses and Dissertations (OATD)**

title:(emirates OR emirati OR emirate OR UAE) OR abstract:(emirates OR emirati OR emirate OR UAE)

**Index Medicus for the Eastern Mediterranean Region (IMEMR)**

emirate emirates emirati UAE

***Appendix A-b*** : Flow Chart of the studies

**Figure 1.** Flow chart of the reference screening and inclusion process

CINAHL: Cumulative Index to Nursing and Allied Health Literature: IMEMR: Index Medicus for the Eastern Mediterranean Region; OATD: Open Access Theses and Dissertations; PQDT: ProQuest Dissertations & Theses Database; IDF: International Diabetes Federation; UAE: United Arab Emirates
