# Supplementary material for: The Epidemiology and Economic Burden of Obesity and Related Cardiometabolic Disorders in the United Arab Emirates: A Systematic Review and Qualitative Synthesis
Source: J Obes. 2018 Dec 3;2018:2185942. doi: 10.1155/2018/2185942 (PMC6311818; doi:10.1155/2018/2185942)
Supplement: Supplementary Materials — Appendix A: search strategy followed and flow chart. Appendix B: quality assessment of the cost of illness for diabetes in the United Arab Emirates study. [file 2185942.f1.zip › Appendix B- Quality assessment of the COI study_JOBE_2499680.docx]

***Appendix B:*** Quality assessment of the cost-of-illness of diabetes in the United Arab Emirates study (Al Maskari et al., 2010)

| **Criterion** | **Yes/Partial/No/Not Applicable*** |
| --- | --- |
| **1. Analytical framework** | |
| a. Is there a defined perspective of the study? | Yes |
| b. Was the appropriate epidemiologic approach taken? | Yes |
| c. Was the study question well specified? | Yes |
| **2. Methodology and data** | |
| a. Was the appropriate method(s) of resource quantification used? | Yes |
| b. Was the resource quantification method(s) valid? | Partial |
| c. Were there appropriate valuation of healthcare resources? | Partial |
| d. Was the approach for valuing indirect costs justified and assumptions valid? | Not Applicable |
| e. Was there appropriate inclusion of intangible costs? | Not Applicable |
| **3. Analysis and reporting** | |
| a. Was the study question addressed in the analysis? | Yes |
| b. Was there a range of cost estimates presented? | No |
| c. Were the main uncertainties identified? | No |
| d. Were sensitivity analyses performed on important parameter estimates, key assumptions and point estimates? | No |
| e. Was sufficient documentation and justification made for cost components, data and sources, assumptions and methods? | Partial |
| f. Was uncertainty of the estimates and its implications sufficiently discussed? | Partial |
| g. Were limitations of the analysis discussed regarding its cost components, data, assumptions and methods? | Partial |
| h. Was there an appropriate degree of detail in the results presented to answer the study question? | Partial |
| ***Percentage (%)*** | ***57.1%*** |
| **Based on the checklist elaborated by Larg & Moss (2011). Percentage of criteria met among applicable ones: Yes= 1; Partial= 0.5; Total score= 8 out of 14 applicable criteria; i.e. the study meets 57% of the listed criteria in the Larg & Moss (2011) checklist.* | |

Larg A, Moss JR. Cost-of-illness studies. Pharmacoeconomics 2011:29:653-71.
